# Ficin–Cyclodextrin-Based Docking Nanoarchitectonics of Self-Propelled Nanomotors for Bacterial Biofilm Eradication

**DOI:** 10.1021/acs.chemmater.3c00587

**Published:** 2023-05-09

**Authors:** Miglė Žiemytė, Andrea Escudero, Paula Díez, María D. Ferrer, Jose R. Murguía, Vicente Martí-Centelles, Alex Mira, Ramón Martínez-Máñez

**Affiliations:** † Genomics & Health Department, FISABIO Foundation, 46020 València, Spain; ‡ Instituto Interuniversitario de Reconocimiento Molecular y Desarrollo Tecnológico (IDM), Universitat Politècnica de València, Universitat de València, València 46022, Spain; § Unidad Mixta UPV-CIPF de Investigación en Mecanismos de Enfermedades y Nanomedicina, València, Universitat Politècnica de València, Centro de Investigación Príncipe Felipe, 46012 València, Spain; ∥ Unidad Mixta de Investigación en Nanomedicina y Sensores, Universitat Politècnica de València, Instituto de Investigación Sanitaria La Fe, 46026 València, Spain; ⊥ CIBER de Bioingeniería, Biomateriales y Nanomedicina (CIBER-BBN), Instituto Carlos III, 28029 Madrid, Spain; # CIBER of Epidemiology and Public Health (CIBERESP), Instituto Carlos III, 28029 Madrid, Spain; ∇ Departamento de Química, Universitat Politècnica de València, Cami de Vera s/n, 46022 València, Spain

## Abstract

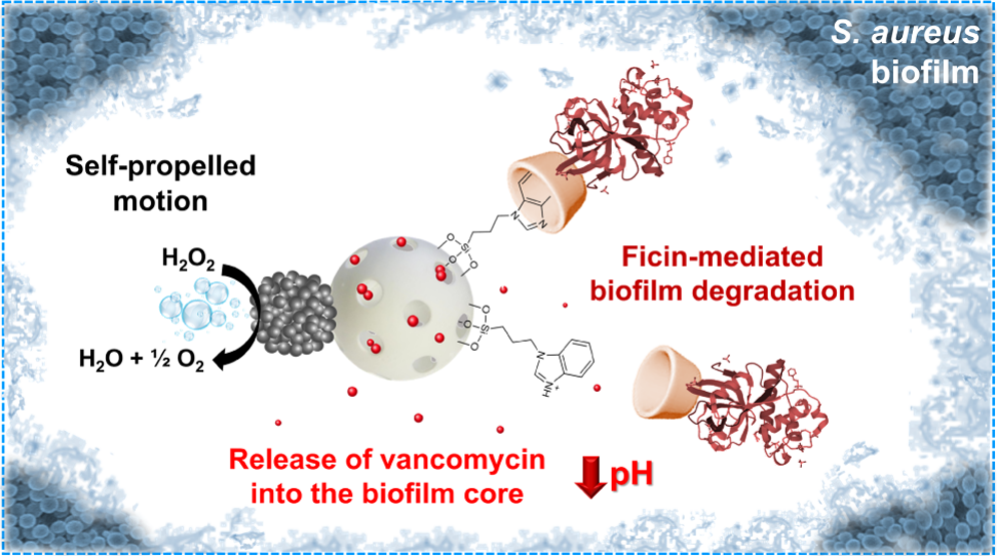

Development of bioinspired nanomotors showing effective propulsion and cargo delivery capabilities has attracted much attention in the last few years due to their potential use in biomedical applications. However, implementation of this technology in realistic settings is still a barely explored field. Herein, we report the design and application of a multifunctional gated Janus platinum–mesoporous silica nanomotor constituted of a propelling element (platinum nanodendrites) and a drug-loaded nanocontainer (mesoporous silica nanoparticle) capped with ficin enzyme modified with β-cyclodextrins (β-CD). The engineered nanomotor is designed to effectively disrupt bacterial biofilms via H_2_O_2_-induced self-propelled motion, ficin hydrolysis of the extracellular polymeric matrix (EPS) of the biofilm, and controlled pH-triggered cargo (vancomycin) delivery. The effective synergic antimicrobial activity of the nanomotor is demonstrated in the elimination of *Staphylococcus aureus* biofilms. The nanomotor achieves 82% of EPS biomass disruption and a 96% reduction in cell viability, which contrasts with a remarkably lower reduction in biofilm elimination when the components of the nanomotors are used separately at the same concentrations. Such a large reduction in biofilm biomass in *S. aureus* has never been achieved previously by any conventional therapy. The strategy proposed suggests that engineered nanomotors have great potential for the elimination of biofilms.

## Introduction

Bacterial infectious diseases have become a global public health problem in recent years, causing elevated morbidity and mortality rates, particularly in immunocompromised patients.^1^ One of the main reasons for this is the microbial ability to adhere to biotic or abiotic surfaces and encase in a self-produced extracellular polymeric substance (EPS) matrix composed of DNA, polysaccharides, and proteins, forming biofilms.^2^ EPS acts as a physical barrier hindering the penetration and diffusion of antibiotics.^3,4^ As a result, the concentration of the drug received by bacteria in biofilms is insufficient for their elimination, which led to an increase in the concentrations of antibiotics used. Related to this is the misuse and overuse of antimicrobials, which are the main drivers in the development of drug-resistant pathogens. In fact, antimicrobial resistance (AMR) is a global health and development threat, the World Health Organization (WHO) having declared that AMR is one of the top 10 global public health threats facing humanity.^5^ In this context, the design of new approaches for the treatment of infections caused by bacteria is of vital importance.

From another point of view, one ambitious objective of nanotechnology is the development of nanodevices able to mimic intrinsic functions of living systems, among which is an autonomous movement. In relation to this topic, synthetic nano/micromotors powered by chemical reactions or external physical forces have attracted much attention recently. In particular, movements in chemically powered nano/micromotors are related to the transformation of chemical substances into mechanical energy. These motors usually contain highly active enzymes^6−8^ or metals with intrinsic catalytic activity, among which platinum^9^ (Pt decomposes hydrogen peroxide in water and oxygen) and zinc^10^ (Zn autooxidation generates hydrogen bubbles) are the most widely used. Regarding the shape, chemical engines are usually based on nanowires,^11^ microtubes,^12^ and Janus-type particles.^13^ Interestingly, the mechanical force generated by the self-propulsion of nano/micromotors can improve penetrability through different barriers. In addition, in relation to biotechnological applications, nano/micromotors with the ability to release therapeutic payloads on command are highly desired. Among materials used in cargo delivery, mesoporous silica nanoparticles (MSNs) can be highlighted due to their biocompatibility, biodegradability, and the possibility to functionalize the external surface with gatekeepers, which allow cargo delivery on command in the presence of selected stimuli.^14^ Although several publications describe the use of antimicrobial-loaded MSNs, these systems did not present self-motion and are mostly focused on the elimination of pathogens in a planktonic state yet not embedded into biofilms.^15^ In this scenario, both characteristics, motion and cargo delivery at-will, could improve the penetration of the antimicrobial therapeutic agent into difficult-to-access areas, making them a powerful tool for active biofilm removal. To date, a few examples describe the use of microengines that are usually tubular in shape and chemically driven,^16−19^ employing high concentrations of H_2_O_2_ (up to 3%) for an effective EPS disruption. In addition, at the nanoscale, Vilela et al. designed drug-free urease-propelled MSN nanomotors for the treatment of *Escherichia coli* (*E. coli*) in urinary tract infections inducing a 60% of biofilm biomass reduction.^8^ In addition, Ji et al.^20^ and Cui and co-workers^21^ designed nanomotors propelled by near-infrared (NIR) light, resulting in a photothermal penetration into the EPS and drug release. However, the described examples still show some drawbacks such as the addition of high levels of toxic chemical fuel, poor mobility, and penetration that does not cause significant physical damage to the EPS or the use of external energy sources (light, magnetism, or ultrasounds) not suitable for treating deep tissue infections. In addition, light-induced photothermal effects may cause collateral damage to adjacent healthy tissues and cells due to the high temperature (≥45 °C) required to achieve complete ablation of pathogens.^22^

**Scheme 1:**
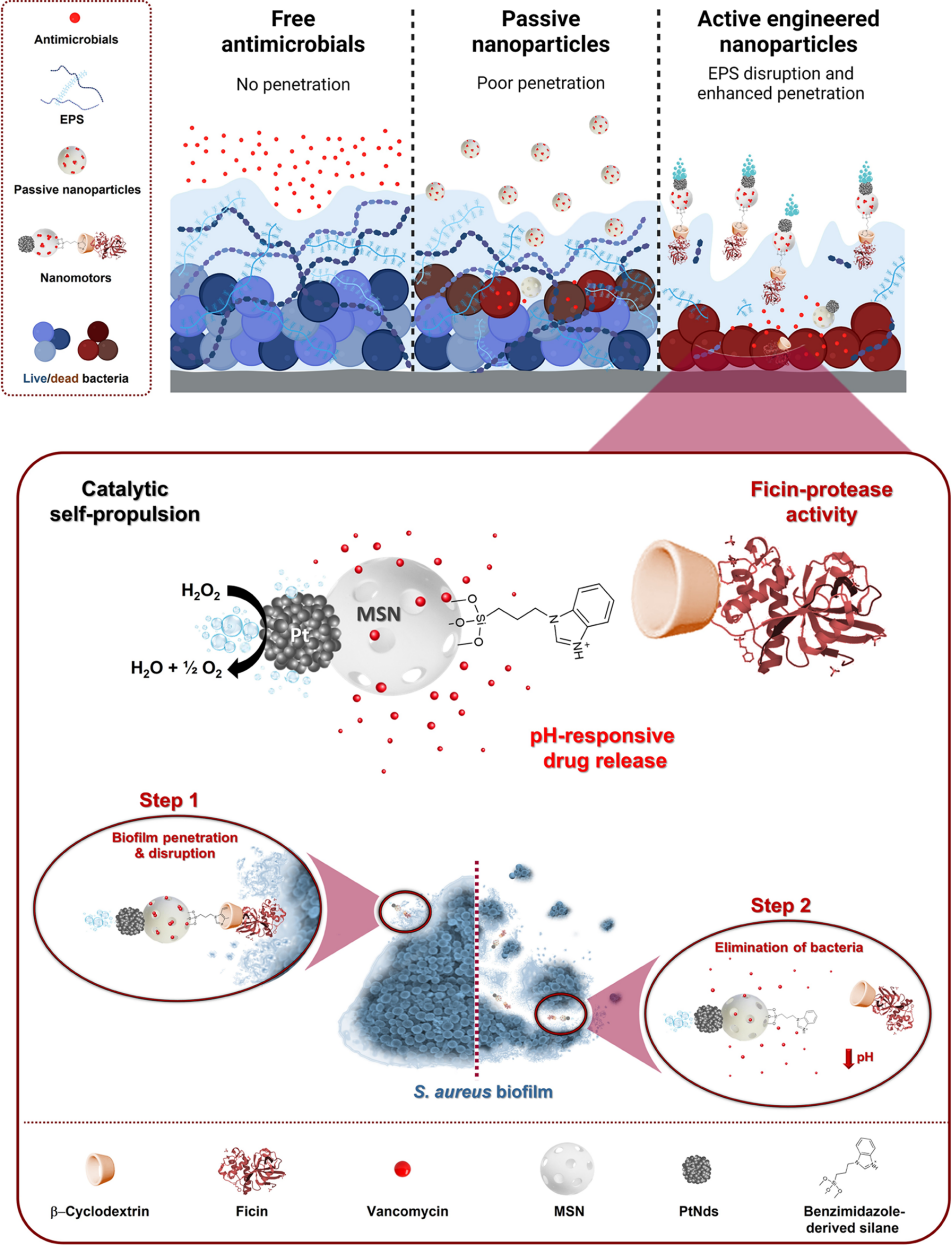
Illustration of Most Representative Strategies to Address Biofilm-Related Infections: Free Antimicrobials, Passive Nanoparticles, and Nanomotors Biofilms are poorly penetrable by antimicrobials and passive nanoparticles, which only remove bacteria from the biofilm surface. In contrast, active nanoparticles penetrate the EPS reaching the deeper layers of the biofilm, increasing the efficacy of antibiotic treatment. The design of our multifunctional H_2_O_2_-fueled Janus Pt–MSN nanomotor is detailed at the bottom of the scheme. Eradication of biofilms is based on two steps: (1) penetration of the nanomotors and disruption of the *S. aureus* biofilm extracellular matrix and (2) elimination of bacteria by pH-triggered controlled release of vancomycin.

Inspired by the above, we report herein the development of chemically fueled Janus Pt–MSN nanomotors capable of effectively penetrating EPS and killing bacterial cells. In particular, we have focused our study on *Staphylococcus aureus*, which is one of the main pathogens responsible for chronic biofilm-associated infections in tissues such as the skin, the respiratory tract, and in medical devices.^23^ Due to the difficulty in preventing the formation and/or eradicating *S. aureus* biofilms, different approaches have been studied, such as the combination of conventional antibiotics,^24^ passive nanoparticles,^15^ or their association with DNAases^25^ or proteases^26^ However, these therapies usually fail to effectively penetrate the intricate EPS of biofilms (Scheme 1, top).

The nanomotor we have designed for biofilm eradication (Scheme 1, bottom) presents two different faces, a mesoporous silica nanoparticle attached to platinum nanodendrites (PtNDs). MSN acts as a nanocontainer for the antibiotic vancomycin. In addition, the MSN face is externally modified with a gating system composed of a pH-sensitive inclusion complex between benzimidazole moieties and β-CD that decorate the protease ficin (a known natural plant protease with unique properties to destroy biofilms)^27^ (EC 3.4.22.3). PtNDs are responsible for self-propelled motion, acting as catalysts for the local decomposition of H_2_O_2_ (l) into H_2_O (l) and O_2_ (g). The vancomycin-loaded ficin-functionalized nanomotor is expected to achieve a deep penetration and disruption of EPS, by the synergistic effect of self-motion and the protease activity of ficin, and effective bacterial killing related to the specific antibiotic delivery inside the acidic pH of the biofilm.

## Results and Discussion

### Nanomotor Synthesis and Characterization

**Figure 1:**
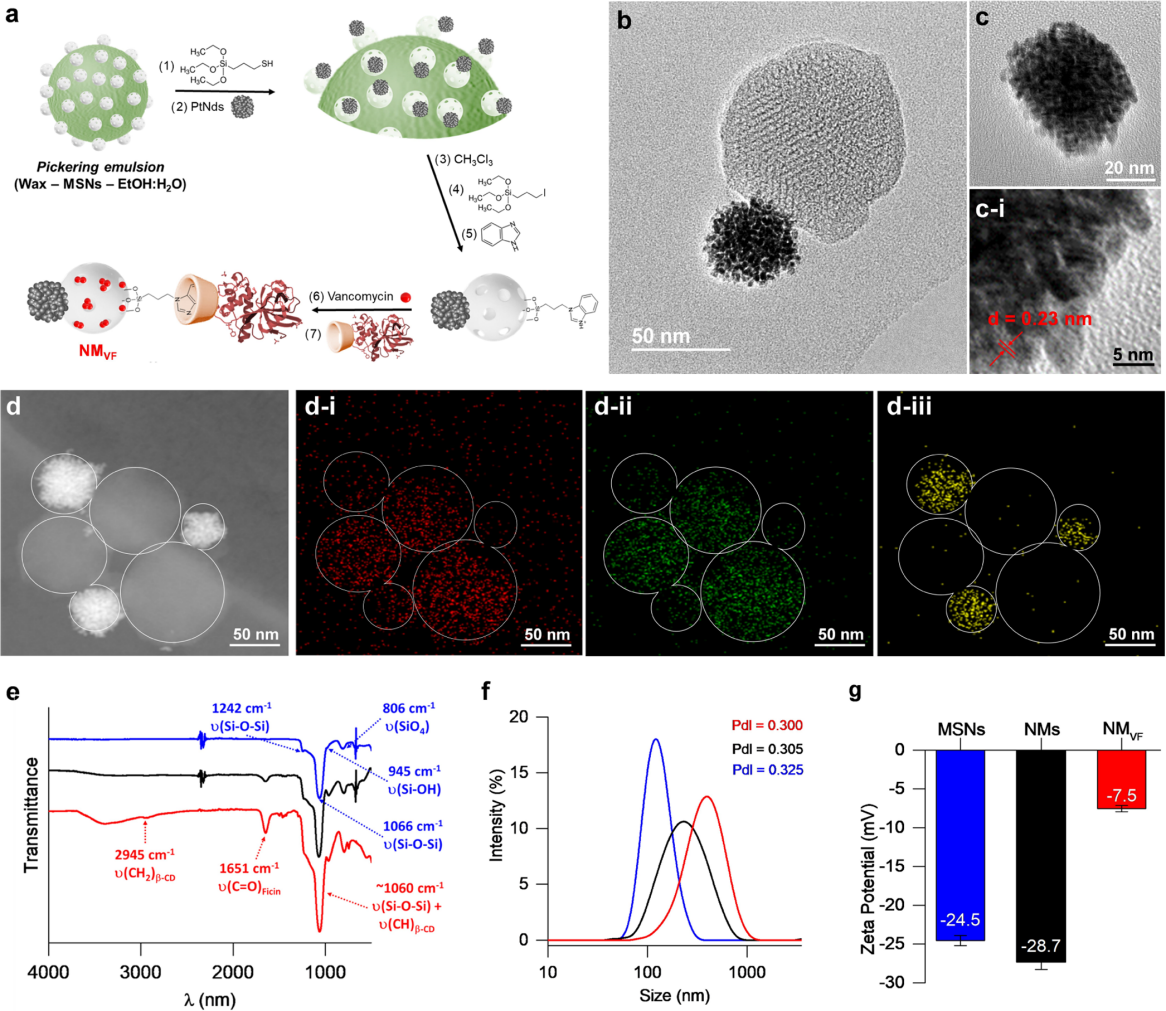
Synthesis and characterization of NM_VF_. (a) Schematic representation of the synthesis process of NM_VF_. (b) HR-TEM image of snowman-like NMs. (c) HR-TEM images of PtNDs (c-i: zoomed image showing the interplanar distance for the (111) plane). (d) Elemental mapping of O_2_ (d-i, red), Si (d-ii, green), and Pt atoms (d-iii, yellow) in NMs. Wt % composition detected: 59.8% of O_2_, 24.3% of Si, and 15.9% of Pt. (e) FTIR spectrum, (f) hydrodynamic diameter distribution (nm), and (g) ζ potential (mV) of MSNs (blue line), NMs (black line), and NM_VF_ (red line).

Nanomotors Janus Pt–MSN (NMs) were made following a synthetic method in which two types of independent nanoparticles, PtNDs and MSNs, were conjugated in a single anisotropic nanodevice (Scheme 1, Figure 1a and Experimental Section).^28,29^ Due to their well-known characteristics, MSNs were selected as cargo nanocontainers, while PtNDs were chosen as the motion system on account of their high surface roughness and catalytic activity toward H_2_O_2_ reduction. MSNs were synthesized by alkaline hydrolysis and condensation of tetraethyl orthosilicate (TEOS) employing cetyltrimethylammonium bromide (CTAB) as the structure-directing agent. Concurrently, PtNDs were synthesized by the autocatalytic chemical reduction of H_2_PtCl_6_ with ascorbic acid in the presence of poly(vinylpyrrolidone) (PVP).^29^ A toposelective synthesis using a Pickering emulsion formed by paraffin wax (oily phase) and water–ethanol (aqueous phase) was used to link both nanoparticles. Briefly, MSNs were partially embedded at the interface of the paraffin/water emulsion allowing the unmasked MSN surface decoration with reactive thiol groups, by reaction with (3-mercaptopropyl) trimethoxysilane. Then, PtNDs were subsequently chemisorbed by thiol bonds. By removing the paraffin with chloroform, the starting Janus nanoparticle NMs were obtained.

To synthesize the final nanomotor (NM_VF_) the MSN face was functionalized with iodopropyl trimethoxysilane to which benzimidazole groups were attached and the mesopores were loaded with the antibiotic vancomycin. Finally, cyclodextrin-modified ficin (F-βCD) was employed as the capping ensemble by the formation of inclusion complexes with the benzimidazole moieties that decorated the MSN surface. Similar nanodevices to NM_VF_ were prepared yet deprived of some of the components, i.e., lacking platinum (NM_MSN_), vancomycin (NM_F_), or ficin (NM_V_) or lacking the Pt motor (MSN_VF_). In addition, a complete nanomotor loaded with rhodamine B-functionalized vancomycin was also prepared (NM_V-RhF_). Studies were also carried out with a system containing in solution all of the unassembled components of the nanomotors (NA). A picture of the nanomaterials used in our study is depicted in Table S1.

The structure and functionalization of the nanomaterials were studied by standard characterization methods. The synthesis of Janus particles, by joining PtNDs and MSNs in a single anisotropic nanodevice, was demonstrated by high-resolution transmission electron microscopy (HR-TEM) (Figure 1b). Figure 1b shows the snowman-like Janus NM morphology with a diameter of 133 ± 7 nm. The efficiency of the Janus Pt–MSN nanoparticle formation was over 61% (Figure S1, mean size ± standard error of the mean, *n* = 160). Individual nanoparticles presented a spherical morphology with an average diameter of 102 ± 13 nm for MSNs and 31 ± 9 nm for PtNDs. In addition, PtNDs are constituted by the union of Pt seeds (Figure 1c), which greatly increases the surface area enhancing the catalytic activity.^30,31^ HR-TEM images also confirmed the typical porous hexagonal arrangement in MSNs, as well as the presence of platinum crystal faces. Figure 1c-i shows an average lattice fringe distance of 2.3 Å, which corresponds to the interplanar spacing for the planes (111) in face-centered cubic (fcc) Pt crystals.^32^ A mapping of the atomic composition of NMs (Figure 1d) by scanning transmission electron microscopy coupled with energy-dispersive X-ray spectroscopy (STEM-EDX) confirmed the composition.

The PXRD spectra of NMs (Figure S2) at high angles show the characteristic Bragg peaks of Pt indexed as (111), (200), and (220) planes, confirming the HR-TEM results. In addition, the spectrum at low angles reveals a diffraction peak at 2.4°, relative to mesoporous structures of type MCM-41 and indexed as a plane (100). This characteristic peak is preserved from NMs to NM_VF_ confirming that surface modification and pore loading do not affect the mesoporous arrangement scaffold. Moreover, N_2_ adsorption–desorption isotherm studies on NMs showed an adsorption step at intermediate values (0.1–0.3 P/P_0_) attributed to the nitrogen condensation inside the mesoporous ordering. Applying the Barret–Joyner–Halenda model (BJH),^33^ NMs’ pore diameter and pore volume are found to be 2.2 nm and 0.3 cm^3^ g^–1^, respectively. The specific surface area of the NMs was calculated by the Brunauer–Emmett–Teller (BET)^34^ model to be 551 m^2^ g^–1^. For NM_VF_ the N_2_ volume adsorbed was dramatically reduced as expected due to the loading with vancomycin and gating functionalization, and a decrease in the pore volume (to nearly zero) and surface area to 5.84 m^2^ g^–1^ was observed (Figure S3 and Table S2).

The construction of NM_VF_ was also studied by FTIR (Figure 1e). The nanomaterials analyzed show the typical bands of siliceous nanomaterials: a band at 806 cm^–1^ attributed to SiO_4_ tetrahedrons, a band at 946 cm^–1^ ascribed to the Si–OH groups, and bands at 1066 and 1242 cm^–1^(shoulder) ascribed to the bond stretching vibrations of Si–O–Si bonds.^35^ The NM_VF_ spectrum shows the bond stretching of CH_2_ groups at 2945 cm^–1^, as well as a broad band at 1060 cm^–1^ corresponding to the cyclic oligosaccharide β-CD. Moreover, the presence of ficin was confirmed by the amide I absorption band at 1651 cm^–1^ and quantified by the bicinchoninic acid assay (BCA) in 18.6 μg per mg of NM_VF_. The amount of β-CD attached to ficin was calculated to be 4 wt % by the phenol–sulfuric acid method. Additionally, the F-βCD content was confirmed by the thermogravimetric analysis and the loaded vancomycin amounted to 22.4 μg per mg of NM_VF_. Contents of the capping unit and cargo were also completed for control nanodevices (Figure S4 and Table S3). In addition, the protein degradation capacity of ficin was evaluated by running a protease activity assay against casein,^36^ with a result of 1.02 × 10^–5^ U mg^–1^ of NM_VF_.

To complete the characterization, the nanomotors were studied by dynamic light scattering analysis (DLS) (Figure 1f,g). The average hydrodynamic diameter augmented from 160 ± 19 nm in MSN to 221 ± 4 nm NMs, reaching a final size of 383 ± 7 nm in NM_VF_, whereas ζ potentials changed from −24.5 and −28.7 mV in MSN and NMs, respectively, to −7.5 mV in NM_VF_ due to the presence of the gatekeeper F-βCD, which has an isoelectric point of 9 and therefore is positively charged at neutral pH.^37^ Both results indicated the success of gatekeeper surface functionalization processes. In addition, DLS studies were also completed for control nanodevices (Figure S5 and Table S4).

### Multifunctional NM_VF_ Capabilities: Controlled Release and Autonomous Motion

#### On-Command Cargo-Controlled Release

**Figure 2:**
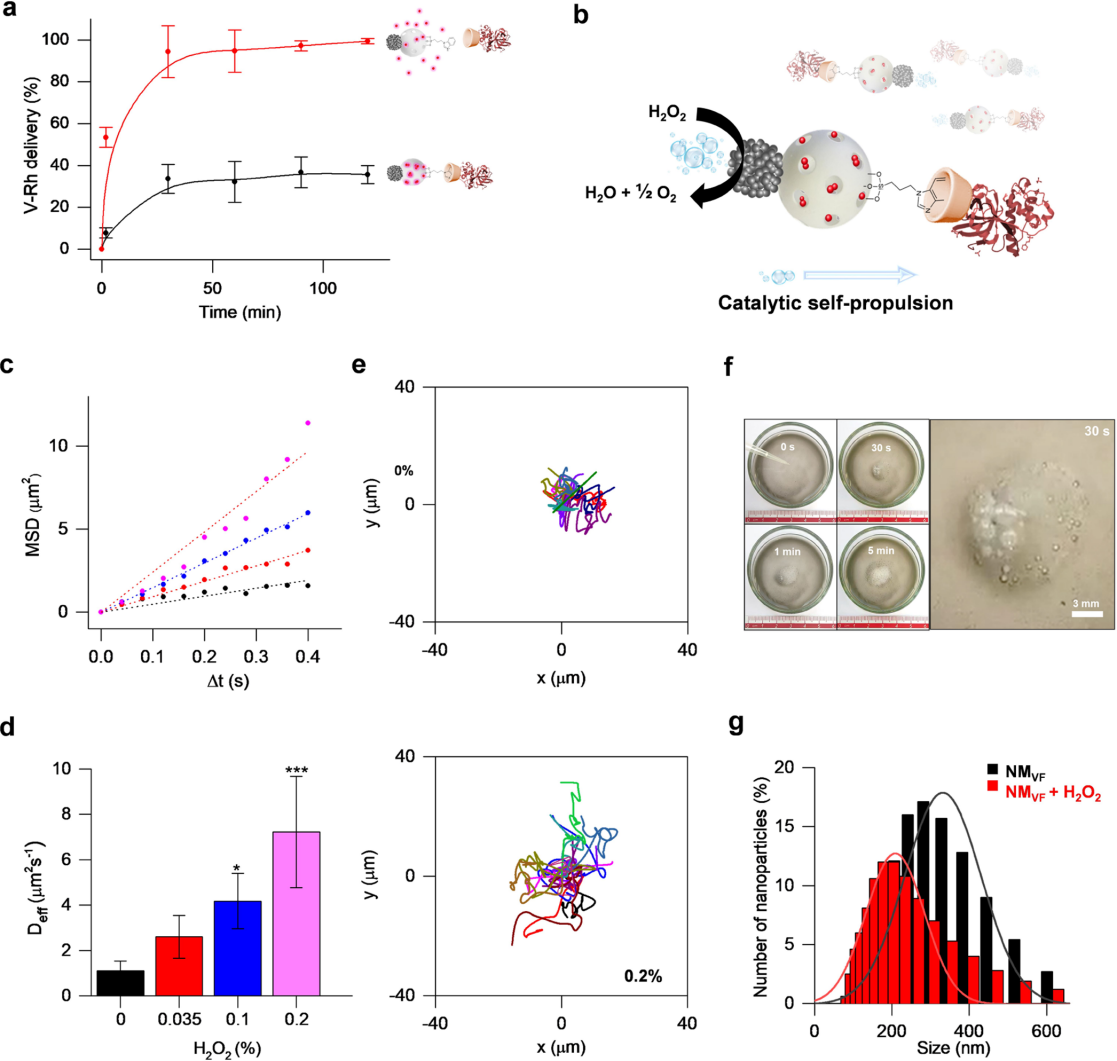
NM_VF_ on-command cargo-controlled release and motion analysis. (a) Normalized controlled release of the loaded V–Rh from NM_V-RhF_ at acidic pH (4.5) (red) and neutral pH (7.5) (black) by measuring Rh B fluorescence at 568 nm, *n* = 3. (b) Schematic illustration of the NM_VF_ autonomous movement by H_2_O_2_ catalytic decomposition on the PtNDs. (c) Mean-square displacement (MSD) vs time interval (Δ*t* = 0.4 s) in the presence of a range of H_2_O_2_ concentrations (0, 0.035, 0.1, and 0.2%). (d) NM_VF_ effective diffusion values (*D*_eff_, μm^2^ s^–1^) obtained from MSD plots for each fuel concentration (error bars represent the standard error of the mean). (e) Representative NM_VF_ tracked spatial trajectories at 0 and 0.2% of H_2_O_2_. (*n* = 15, one-way ANOVA and Dunnett’s multiple comparison test, *p < 0.05, ***p < 0.0001). (f) Time-lapse images of NM_VF_ catalytic decomposition of 0.15% H_2_O_2_. Bubble generation can be observed by the naked eye in only 30 s. (g) Hydrodynamic diameter distribution (nm) of NM_VF_ in the absence (black) and presence of 0.2% H_2_O_2_ (red).

After the characterization of the nanodevices, we tested the nanomotor ability to deliver vancomycin in acidic conditions mimicking the *S. aureus* biofilm (ca. pH 5).^38^ For these studies, nanomotors loaded with vancomycin modified with rhodamine B (V–Rh), i.e., NM_V-RhF_, were used (Figure S6). Nanodevices were dispersed in sodium phosphate buffer (PBS) or in acidic acetate buffer and the fluorescence signal from delivered V–Rh was monitored at scheduled times. At neutral pH, there is no significant cargo release to the medium, whereas, at acidic pH, maximum cargo delivery occurs in only 30 min (Figure 2a). Preferential cargo delivery triggered by acidic pH is due to the protonation of the benzimidazole groups (p*K*_a_ = 5.55)^39^ that results in a disassembly of the benzimidazole and F-βCD inclusion complex. The benzimidazole and β-CD complex formation constant is reduced from 104 ± 8 M^–1^ (*K*_βCD-Bnz_) for neutral benzimidazole to 42 ± 12 M^–1^ (*K*_βCD-Bnz+_) for the protonated benzimidazole form.^40^

#### Motion Analysis

Previous to characterizing the nanomotor motion, the catalytic activity of PtNDs in NMVF was demonstrated following a spectrophotometric assay based on the oxidation of 2,2′-azino-bis-(3-ethylbenzthiazoline-6-sulfonic acid) (ABTS) in the presence of H_2_O_2_. The reaction rate of ABTS oxidation by PtNDs in NM_VF_ at different H_2_O_2_ concentrations displayed a typical Michaelis–Menten behavior (Figure S7), demonstrating that the nanomotor exhibits a peroxidase-like activity, with an apparent affinity value (*K*_M_, eq 1) for H_2_O_2_ of 14.95 mM, a higher value than the *K*_M_ of native horseradish peroxidase (4.37 mM).^41^

The self-propelled motion of the nanomotor NM_VF_ based on the catalytic decomposition of H_2_O_2_ (Figure 2a) was investigated by different techniques. First, it was characterized by nanoparticle tracking analysis (NTA) using a Nanosight NS300 instrument. The trajectories of nanomotors were recorded in real time in the presence and absence of the fuel H_2_O_2_ (concentrations from 0 to 0.2%) in PBS. For each sample, 30 s videos were registered and analyzed. The *x*–*y* coordinates of 15 nanoparticles (sizes between 100 and 400 nm) were used to obtain NM_VF_ mean-square displacement (MSD) using eq 2. MSDs of NM_VF_ were plotted vs time intervals (Δ*t*) in each fuel concentration to evaluate motion behavior, through an in-house developed R code. Results, summarized in Figure 2c, reveal that MSD presents a linear correlation with Δ*t* for all H_2_O_2_ concentrations tested. Applying eq 2 to these data, the diffusion coefficients (*D*) were obtained (Figure 2d) expressed as *D*_0_ for nonfueled nanomotors and the effective diffusion coefficient (*D*_eff_) for H_2_O_2_-fueled nanomotors. Nonfueled nanomotors display a *D*_0_ of 1.1 ± 0.4 μm^2^ s^–1^, which is close to the theoretical value (1.1 μm^2^ s^–1^), predicted by the Stokes–Einstein equation (eq 3) for nanoparticles of the same size, 383 nm. In contrast, *D*_eff_ augments from 2.6 ± 0.9 μm^2^ s^–1^ at 0.035% H_2_O_2_ to 7.2 ± 2.4 μm^2^ s^–1^ at 0.2% H_2_O_2_, which demonstrates a fuel concentration-dependent enhanced diffusive motion. This diffusive increase is also supported by the trajectories of fueled nanomotors in comparison with unfueled particles (Figures 2e and S8). These findings indicate that NM_VF_ acts as an autonomous catalytic nanomotor with remarkable diffusion coefficients in the presence of relatively low H_2_O_2_ concentrations. Enhanced effective motion is attributed to the high density of active sites and surface roughness of PtNDs, as well as to the asymmetry of the nanodevice NM_VF_ that presents two very differentiated faces, i.e., PtNDs and MSNs. On the other hand, the propulsion mechanism followed by the NM_VF_ is based on the periodic formation, growth, and collapse of O_2_ bubbles.^42−45^ In fact, O_2_ bubbles were observed by the naked eye after only 30 s of fuel addition (Figure 2f). Furthermore, the NM_VF_ motion was evaluated by DLS in the absence and presence of 0.2% H_2_O_2_. The results show that H_2_O_2_-driven NM_VF_ exhibited approximately 200 nm reduction in the apparent size compared to nonfueled NM_VF_ (Figure 2g). This agrees with the *D*_eff_ values obtained since the size and diffusion are inversely related according to the Stokes–Einstein equation. Finally, the autonomous motion of NM_V-RhF_ in the presence of *S. aureus* cells was corroborated by confocal laser scanning microscopy (CLSM) (Video S1), noting at this point that due to the resolution of the microscope, aggregations of nanomotors are shown.

Overall, these studies confirmed that our developed NM_VF_ contain active ficin protease and are capable of (i) vancomycin delivery triggered by acidic pH conditions simulating an *S. aureus* biofilm interior environment and (ii) displaying autonomous motion at low H_2_O_2_ concentrations. Accordingly, NM_VF_ complies with ideal features, for being a self-propelled antibiotic vehicle for the treatment of biofilms.

### Eradication of Preformed and Mature Biofilms

**Figure 3:**
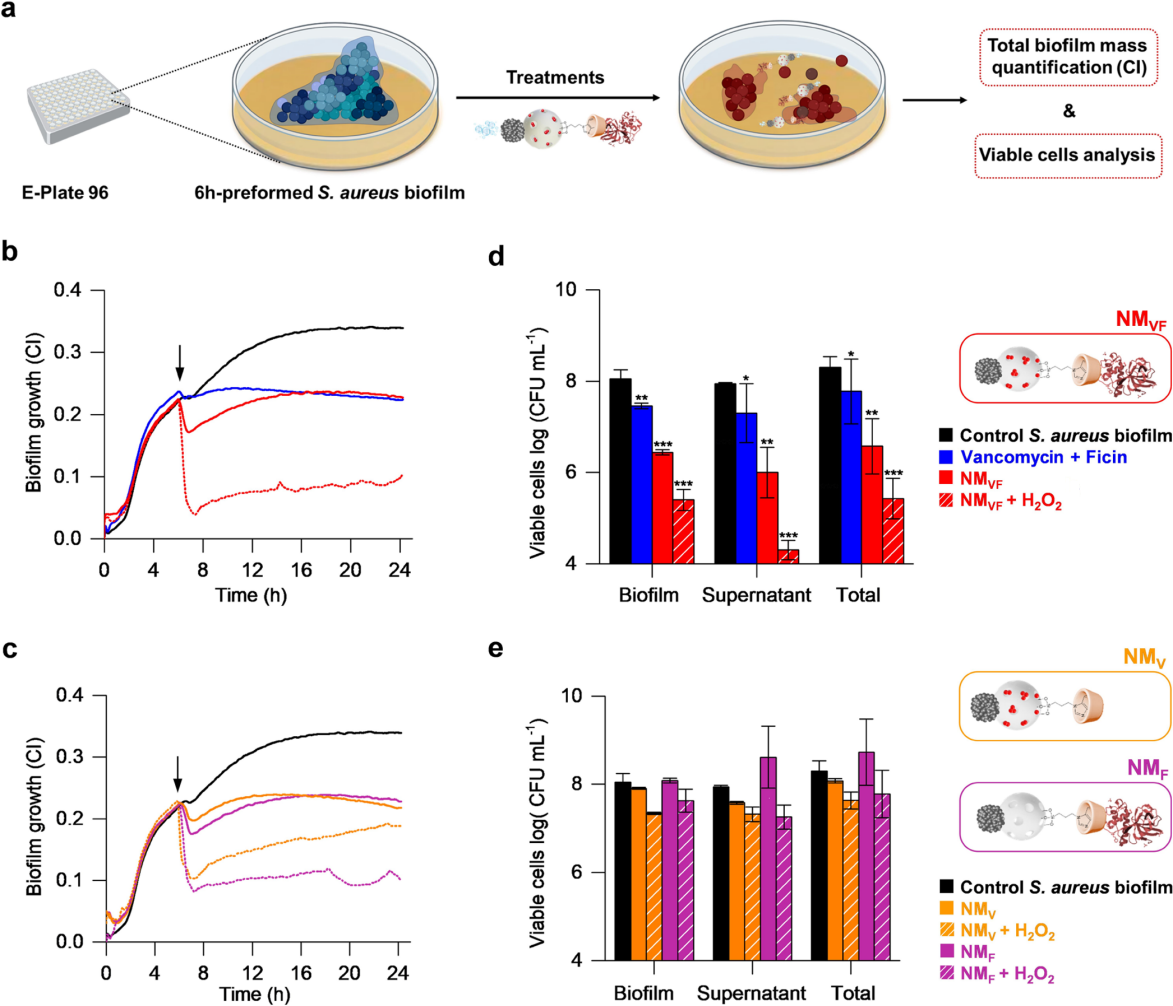
Antimicrobial capacity of NM_VF_ in preformed 6 h *S. aureus* biofilms. (a) Schematic representation of the experimental procedure followed to evaluate the effect of NM_VF_ and control nanodevices on preformed *S. aureus* biofilm growth. (b) Biofilm growth, as quantified by impedance measurements after treatment with free VF (blue), nonfueled NM_VF_ (red), and NM_VF_ in the presence of 0.15% H_2_O_2_ (red striped), (data presented as the mean, *n* = 3). (c) Biofilm growth, as quantified by impedance measurements after treatment with control nanodevices, NM_V_ (orange) and NM_F_ (purple), in the absence and presence of 0.15% H_2_O_2_ (striped), (data presented as the mean, *n* = 3). (d) and (e) Bacterial cell viability in biofilms and supernatants after the same treatments, expressed as log (CFU mL^–1^) ± standard error of the mean (SE). Statistically different CFU counts were assessed at 24 h or growth. *T*-test values, * *p* < 0.05, ** *p* < 0.01, ***p < 0.001. (Treatments were added at 6 h. Concentrations: 1 mg mL^–1^ nanodevices, 23 μg mL^–1^ V, and 25 μg mL^–1^ F).

Encouraged by previous results, the effect of nanomotor NM_VF_ fueled by H_2_O_2_ on *S. aureus* biofilms was studied. Previously it was confirmed that although high fuel concentrations are slightly harmful to *S. aureus* bacteria, biofilm growth and viability are not affected at low H_2_O_2_ concentrations (Figure S9). Based on these studies, a 0.15% H_2_O_2_ concentration was selected for antibiofilm assays. Biofilms were cultivated for 6 h and then either treated with 1 mg mL^–1^ NM_VF_ in the absence or presence of 0.15% H_2_O_2_ or with the combination of free vancomycin (23 μg mL^–1^) and ficin (25 μg mL^–1^) (VF), (Figure 3a). The growth of preformed *S. aureus* biofilms was measured by real-time impedance analysis in xCELLigence equipment and expressed as cell index (CI) values, which correlate with the total biofilm mass.^46^ Administration of nonfueled NM_VF_ only caused a 34% decrease in the total biofilm mass at 24 h of biofilm growth, a value similar to that obtained with the VF treatment. On the contrary, treatment with H_2_O_2_-fueled NM_VF_ caused an 82% biofilm disruption in only 1 h, and up to 72% at 24 h, compared to the untreated control (Figure 3b). The initial larger effect at 1 h is likely due to the breakdown of the biofilm structure when the nanodevice movement is maximal, as a result of an optimal concentration of H_2_O_2_ and optimal activity of the antibiotic. Given that not all bacteria are killed and that some of them are released into the supernatant, the lower concentration of the active antibiotic and the lower movement of the particles with time will allow some viable bacteria to attach again to the biofilm, leading to a slightly lower effect at 24 h. At this point, it is noteworthy to remark that such dramatic biofilm biomass reduction has never been achieved for any conventional therapy against *S. aureus*.^47^ Additionally, the effect of incomplete nanomotors, NM_V_ or NM_F_, on the biofilm disruption was also evaluated. Biofilm growth results (Figure 3c) indicate that both control nanodevices were able to reduce preformed biofilms to a similar extent compared to the free VF treatment (34% biomass reduction) in the absence of H_2_O_2_. In addition, when both nanodevices were activated with 0.15% H_2_O_2_, biofilm biomass was reduced up to 44% for NM_V_ and 68% for NM_F_, respectively. As expected, biofilm biomass reduction mediated by control NM_F_ was similar to the result of NM_VF_, since both present the same EPS disruptive elements (autonomous movement and ficin protease activity). Biofilm growth studies were also performed for free vancomycin (V) and free ficin (F) with nonrelevant results compared to treatment with fueled NM_VF_ (Figure S10a).

After confirming the efficient EPS disruption by the fueled NM_VF_ nanomotor, the next step was the evaluation of the antimicrobial effect resulting from the controlled release of vancomycin. For this purpose, the number of bacteria was determined by viable colony counts in both the remaining biofilm and the supernatant. VF treatment showed a low capacity (<1 log decrease) to reduce cell viability in the supernatant, which was even weaker in bacteria embedded in the biofilm (*p*-values<0.01). On the other hand, the total cell viability was reduced in both the biofilm and supernatant by almost 2 orders of magnitude when biofilms were treated with NM_VF_ in the absence of fuel (*p*-value < 0.001). In contrast, NM_VF_ activation with 0.15% H_2_O_2_ led to efficient killing of both biofilm-embedded and free-floating bacterial cells, reaching over 3-logs reduction in viable cells compared to the untreated control (*p*-value < 0.001), (Figure 3d). This suggests that the presence of ficin in the nanodevice and controlled cargo release under acidic pH can result in a potentiated effect of vancomycin, killing of both biofilm-embedded and free-floating *S. aureus* cells. Likewise, the biocidal effect of incomplete nanomotors, NM_F_ or NM_V_, was evaluated (Figure 3e). NM_F_ in the presence of H_2_O_2_ did not produce a significant reduction of cell viability, neither in the biofilm nor in the supernatant (*p*-value of total viable cells >0.1). This suggests that, although the movement of the nanomotor coupled with its functionalization with ficin presents a strong ability to detach the biofilms (as shown by CI impedance results above), NM_F_ was not able to kill the bacteria embedded in the biofilm. Similar results were observed for NM_V_, showing that in the absence of ficin nanomotors fail to disrupt the biofilm. As a consequence, NM_V_ did not reach the acidic area and did not release the antibiotic, so the bacterial cell viability remains similar to the control. Finally, we also tested the effect of free V and F (Figure S10b), the control MSN_VF_ and the unassembled nanodevice, NA, on cell viability (Figure S11). Results showed no relevant antimicrobial properties both in the presence or the absence of fuel. Taken together, these studies demonstrate that NM_VF_ is adequate for biofilm disruption and bacterial elimination, whereas, the lack of elements in the nanodevice results in a lower performance.

**Figure 4:**
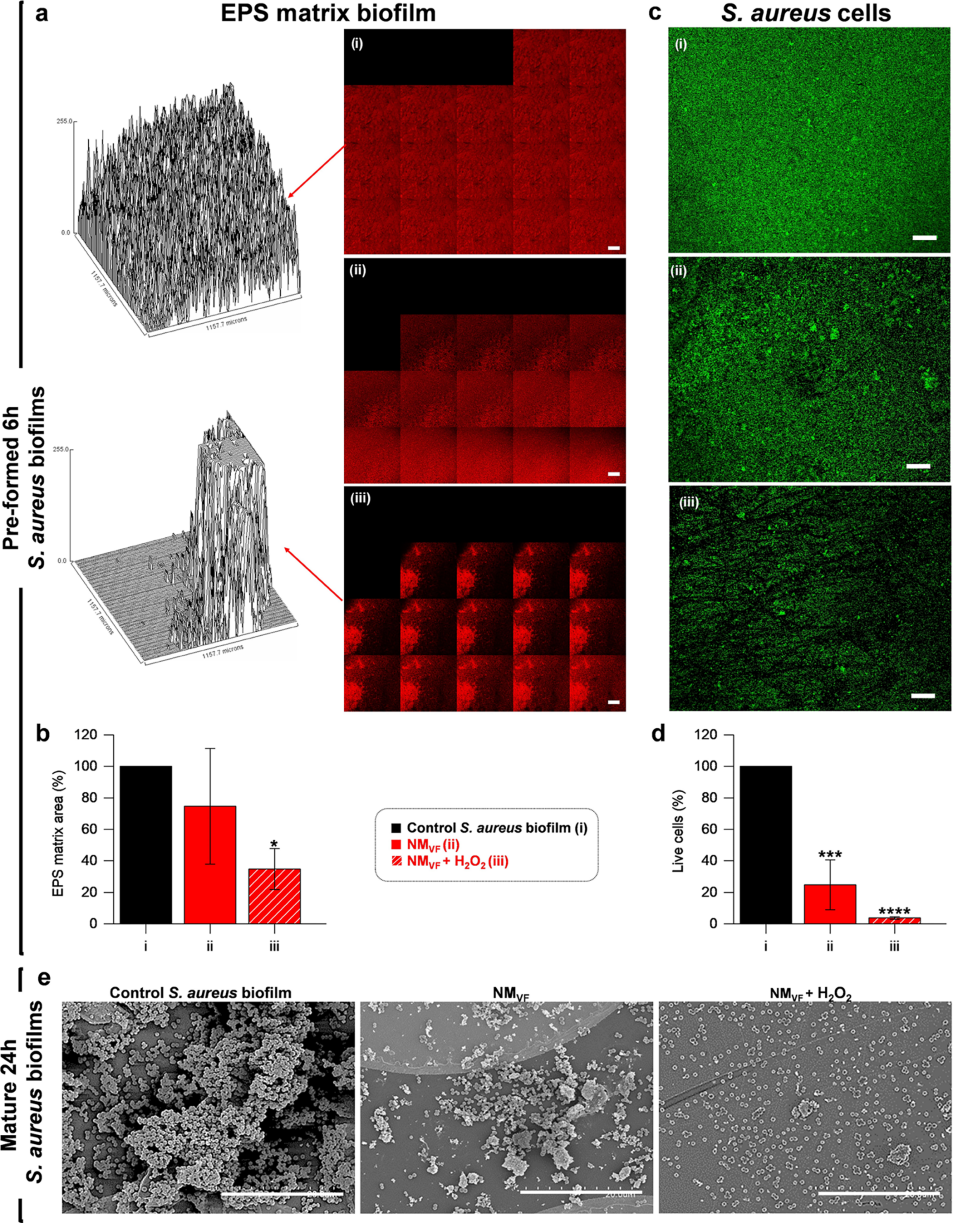
Disruption of EPS and live cells present in preformed 6 h *S. aureus* biofilms after treatment with H_2_O_2_-fueled NM_VF_. Experimental groups were the untreated control (i), 1 mg mL^–1^ NM_VF_ (ii), and 1 mg mL^–1^ NM_VF_ fueled with 0.15% H_2_O_2_ (iii). (a) CLSM analysis of *S. aureus* biofilms labeled with an Alexa Fluor 680-dextran conjugate after 24 h of treatment. Each of the 20 images shows the area covered by EPS (in red) in the biofilm stacks, taken from the top to the bottom. On the left is shown a 3D reconstruction of the top layer biofilm of the control (i) and fuel-activated NM_VF_ (ii) treatment at 24 h. Biofilm reconstructions were performed by ImageJ software. (b) Quantification of matrix glucan in the top layer of (i)–(iii) treatments, expressed as the area percentage ± SE at 24 h. (c) CLSM images showing viable cells stained with SYTO9 (green) remaining after 24 h of treatments (i)–(iii). (d) Fluorescence-based quantification of live cells, expressed as the percentage ± SE regarding the control. Areas covered by the matrix and live cells′ fluorescence intensity were estimated by ImageJ software (*n* = 3, one-way ANOVA and Dunnett’s multiple comparison test, *p < 0.05, ****p < 0.0001). Scale bar: 200 μm. (e) SEM micrographs of 24 h mature *S. aureus* biofilms. (i) Untreated control, (ii) biofilm treated with an NM_VF_ in the absence of H_2_O_2_, iii biofilm treated with NM_VF_ and fueled with H_2_O_2_. Scale bar: 20 μm.

In addition, to visualize the effects of NM_VF_ on biofilms, the EPS of the preformed 6 h *S. aureus* biofilm was labeled using an Alexa Fluor 647-dextran conjugate and then the biofilm was treated with 1 mg mL^–1^ NM_VF_ or with 1 mg mL^–1^ NM_VF_ fueled with 0.15% H_2_O_2_. A significant reduction in the area covered by the EPS matrix (labeled in red) was observed after the treatment with H_2_O_2_-fueled NM_VF_, whereas the nonfueled NM_VF_ did not show a significant effect. The EPS degradation by NM_VF_ in the presence of H_2_O_2_ is clearly observed in the three-dimensional (3D) reconstruction of the biofilm top layer using ImageJ software (Figure 4a). Compared to the untreated control, fueled NM_VF_ produced a 66% reduction in the EPS area of the top layer (Figure 4b), in agreement with the previous CI analysis (*vide ante*).

In addition, the percentage of viable bacterial cells in *S. aureus* biofilms was also quantified by CLSM. In this case, preformed *S. aureus* biofilms were incubated as above with 1 mg mL^–1^ NM_VF_ or with 1 mg mL^–1^ NM_VF_ fueled with 0.15% H_2_O_2_ for 24 h and then stained with SYTO9 green (Figure 4c). Image analysis showed a significant decrease in live cells (labeled in green) in both samples, treated with 1 mg mL^–1^ NM_VF_ both in the presence and absence of H_2_O_2_ when compared to the control group (Figure 4d). Remarkably, H_2_O_2_-fueled NM_VF_ produced a 96% decrease in viable cells, compared to the untreated control. In addition, it was confirmed that 0.15% H_2_O_2_ did not have a significant effect on EPS disruption or cell viability in the biofilm (Figure S13).

To complete the in vitro study, we further tested the ability of the nanomotor to eradicate mature *S. aureus* biofilms by scanning electron microscopy (SEM) after 24 h of treatment. SEM images showed that both H_2_O_2_-fueled and nonfueled NM_VF_ reduced biofilm biomass, yet the larger EPS destruction and the lowest levels of bacterial aggregates were obtained for H_2_O_2_-fueled NM_VF_ (Figure 4e). The combination of free V and F also reduced biofilm biomass but far less than H_2_O_2_-fueled NM_VF_ (Figure S13).

In summary, our results highlight that the NM_VF_ nanodevice loaded with vancomycin and capped with ficin is able to destroy the complex EPS matrix of preformed and mature *S. aureus* biofilms and kill bacteria at low fuel concentrations. The combination of synergistic capabilities (movement, vancomycin, and ficin) in a single multifunctional nanodevice improves the results of previously developed nano/micromotors focused on the same application.

## Conclusions

Biofilm eradication is a key issue in the successful treatment of many severe infectious diseases. In addition, elimination of biofilms on surfaces used in the food industry and in medical devices is critical. However, nowadays this goal is not efficiently achieved with common approaches. In fact, the only treatment of biofilm-related infections in medical devices that cannot be surgically removed requires the administration of high dosages of antibiotic combinations that may lead to multidrug resistance, failure of different organs, or even death. These issues have fueled the research of new concepts for biofilm eradication, a problem of significant importance.

In an attempt to contribute to this area, we report herein the preparation of multifunctional Janus Pt–MSN nanomotors as a new approach for the eradication of biofilms. In the nanomotor, MSNs are selected as cargo nanocontainers, while PtNDs are used as the motion system considering the catalytic Pt ability to reduce H_2_O_2_. The pores in the MSN face are loaded with the antibiotic vancomycin and capped with inclusion complexes between benzimidazole groups, decorating the MSN surface, and CD-functionalized ficin enzyme. Vancomycin is a common strategy to treat biofilm-related infections caused by S. aureus. However, strains sensitive to this antibiotic show extreme resistance when the biofilm is already formed,^45^ resulting in the emergence of vancomycin-resistant strains.^46^

The nanomotor NM_VF_ synergistically combines three strategies in one single nanoparticle to disrupt biofilm EPS and kill bacteria: (i) self-propelled motion due to the catalytic decomposition of H_2_O_2_, (ii) ficin ability to degrade the extracellular polymeric matrix of the biofilm, and (iii) controlled pH-triggered vancomycin delivery at the acidic pH of the biofilm. Our engineered NM_VF_ nanomotor exhibits an autonomous catalytic motion in response to H_2_O_2_, displaying a remarkable increase in the diffusion coefficient from 1.11 ± 0.42 μm^2^ s^–1^ for nonfueled nanomotors to 7.22 ± 2.45 μm^2^ s^–1^ at low concentrations of fuel (0.2%). In addition, NM_VF_ allows controlled delivery of vancomycin at acidic pH (inside the biofilm) due to protonation of the benzimidazole groups, which results in disassembly of the benzimidazole and F-βCD inclusion complex. On the other hand, antimicrobial studies on the real-time growth of *S. aureus* biofilms treated with H_2_O_2_-activated NM_VF_ demonstrate an 82% disruption of the total biofilm mass in only 1 h. As far as we are aware, this drastic reduction in biomass has never been achieved with any conventional therapy against *S. aureus*.^48,49^ Remarkably, activated NM_VF_ exhibits highly effective biocidal activity, resulting in 96% reduction in viable bacterial cells embedded in the biofilm as a result of the controlled release of the antibiotic. This behavior contrasts with that shown by NM_VF_ in the absence of fuel or when noncomplete nanomotors or free vancomycin or ficin were used. In these cases, biofilm disruption, as well as bacterial killing, is much less effective.

Overall, the *in vitro* results exposed in the current manuscript may provide a long-sought solution to the problem of how to approach the treatment of infections caused by bacterial biofilms. Although we are aware that the external addition of the fuel might limit some in vivo applications, our nanomotors powered with low concentrations of H_2_O_2_ might find application in certain microenvironments. These would include tissues in which endogenous H_2_O_2_ is present or released, such as those that occur in the wound inflammatory response,^50^ or in vaginal infections, where the presence of H_2_O_2_-producing *Lactobacillus*(51) would facilitate cargo release from the nanodevice. Self-propelled nanoparticles could also be used to treat oral infections caused by opportunistic pathogens such as different *Candida spp*, with the aid of H_2_O_2_ mouth rinses. Furthermore, it is well known that H_2_O_2_ is widely used as a potent surface disinfectant in diverse areas, such as the medical and alimentary industries, opening the possibility of applying engineered nanomotors as improved sterilizing methods in these fields. In addition to surface sterilization, nanodevices could be used as a new approach to sterilize indwelling medical devices before their implantation in the human body in order to prevent biofilm formation and infection risk, especially in immunocompromised patients.^52^ In addition, the nanomotor design presented here is very versatile, allowing external functionalization with other biomolecules and/or loading with different antibiotics. Therefore, the described strategy could be easily extrapolated to treat biofilms of other species. These would include the elimination of harmful opportunistic pathogens such as *Pseudomonas aeruginosa*, *Listeria monocytogenes*, *Enterococcus faecalis*, and others that are highly recalcitrant to common disinfectants.^53^ We hope that the proof of concept of this triple-strategy combination stimulates further research in the field by the implementation of alternative self-propelling fuels, antimicrobial cargos, and enzymes, and the validation of the system in relevant environments.

## Experimental Section

### Chemicals

*n*-Cetyltrimethylammonium bromide (CTABr), tetraethyl orthosilicate (TEOS), dihydrogen hexachloroplatinate (H_2_PtCl_6_), poly(vinylpyrrolidone) (PVP), ascorbic acid, paraffin wax, (3-mercaptopropyl)trimethoxysilane, (3-iodopropyl)trimethoxysilane, benzimidazole (BENZ) triethylamine (TEA), vancomycin (C_66_H_75_Cl_2_N_9_O_24_), ficin from fig tree latex, *N*-(3-dimethylaminopropyl)-*N*′-ethylcarbodiimide hydrochloride (EDC), *N*-hydroxysuccinimide (NHS), β-cyclodextrin (β-CD), rhodamine B (Rh), 2,2′-azino-bis(3-ethylbenzothiazoline)-6-sulfonic acid (ABTS), trifluoroacetic acid (TFA), and casein from bovine milk were purchased from Sigma Aldrich. Alexa Fluor 680-dextran conjugate, bicinchoninic acid assay (BCA) kit, hydrogen peroxide (30%, v/v), and SYTO9 were supplied by Thermo Fisher. Sodium hydrogen phosphate monohydrate, disodium hydrogen phosphate heptahydrate, sodium acetate, sodium hydroxide (NaOH), ethanol, toluene, chloroform, dimethyl sulfoxide (DMSO), acetonitrile, phenol, sulfuric acid, and acetic acid were provided by Scharlau. Tryptic Soy Agar plates (TSA) and Tryptic Soy Broth (TSB) were used in this study.

### General Methods and Instruments

Powder X-ray diffraction (PXRD) analysis was carried out using a D8 Advance Seifert 3000TT diffractometer using Cu Kα radiation at low angles (1.5 ⟨2θ⟩ 7, with steps of 0.04° and 3s for a step) and high angles (35 ⟨2θ⟩ 80 with steps of 0.04° and 1s for a step). N_2_ adsorption–desorption isotherms were recorded using a Micromeritics TriStar II Plus automated analyzer. Nanomotors were degassed at 90 or 120 °C under vacuum overnight. The specific surface area was calculated from the adsorption data within the low-pressure range using the Brunauer–Emmett–Teller (BET) model, while the pore size was determined following the Barrett–Joyner–Halenda (BJH) method. Thermogravimetric analysis (TGA) was performed with a TA Instruments SDTQ600 apparatus in an oxidizing atmosphere (air, 80 mL min^–1^) and a heating rate program between 393 and 1273 °C at 10 °C min^–1^ followed by an isothermal heating step at 1273 °C for 30 min. Fourier transform infrared spectroscopy (FTIR) measurements were performed in a Tensor 27 instrument (Bruker). Dynamic light scattering (DLS), and ζ potential evaluations were carried out using a Zetasizer Nano ZS (Malvern). Nanoparticle tracking experiments were performed using a Nanosight NS300 (Malvern). UV–visible measurements were performed with a JASCO V-650 spectrophotometer. Fluorescence measurements were carried out in a JASCO FP-8500 spectrophotometer. Elemental mapping was conducted using scanning transmission electron microscopy coupled with electronic energy-dispersive X-ray spectroscopy (STEM-EDX) using a JEM 2100F instrument. Nanomotor transmission electron microscopy (TEM) images were achieved using a JEOL TEM-2100F electron microscope. Optical density (OD) measurements were performed with a Tecan spectrophotometer (Durham, NC). Bacterial cell index experiments were realized with an xCELLigence real-time analyzer (Agilent). Confocal microscopy imaging was obtained with a Leica TCS SP8 AOBS inverted laser scanning confocal. Scanning electron microscopy images of *S. aureus* biofilms were performed using a Hitachi S-4800 field emission scanning electron microscope (Electron Microscopy Service Valencia University, Spain).

### Synthesis of Nanoparticles

#### Mesoporous Silica Nanoparticles (MSNs)

1 g of CTABr was dissolved in 480 mL of deionized water. Then, 3.5 mL of 2 M NaOH was added to the mixture, and the temperature was set at 80 °C. Then, 5 mL of TEOS was added dropwise to the solution that was stirred for 2 h, giving a white precipitate as a result. Next, the solid product was isolated by centrifugation, washed with deionized water, and dried at 70 °C. Finally, the solid was calcinated for 5 h at 550 °C under an oxidant atmosphere, to obtain the final nanoparticles, MSNs.

#### Platinum Nanodendrites (PtNDs)

164 mg of H_2_PtCl_6_ and 20 mg of PVP were mixed in 20 mL of deionized water. Concurrently, 350 mg of ascorbic acid was dissolved in 10 mL of deionized water. This mixture was poured dropwise to the H_2_PtCl_4_ solution. Then, the temperature was increased at 45 °C and the reaction was magnetically stirred for 3 h. The color change, from pale yellow to black reveals the successful synthesis of platinum nanodendrites, PtNDs.^54^

#### Janus Pt–MSN Nanomotors (NMs)

The synthesis of Janus Pt–MSN was performed following a method previously described by us.^28,29^ 180 mg of MSNs was dispersed in 10 mL of a 6.7% ethanol solution in water. 208 μL of CTAB (1 μM) was added and the temperature was set at 75 °C. Then, 1 g of paraffin wax was added. Once the paraffin was melted, an Ultra-Turrax T-8 (IKA) was used for 10 min to homogenize the sample. Later, the reaction was magnetically stirred for 1 h at 75 °C to create a Pickering emulsion. To the resultant cooled mixture, 200 μL of (3-mercaptopropyl) trimethoxysilane, and 10 mL of methanol were added. Then, the mixture was magnetically shaken for 3 h. Next, the obtained solid was isolated by centrifugation and washed with methanol twice. Afterward, the one-face mercapto-functionalized MSNs reacted with the previously synthesized PtNDs, under magnetic stirring at room temperature overnight, to yield the final Janus Pt–MSN nanomotors, NMs, the product was filtered, washed with chloroform, and dried.

#### NM/MSN Surface Functionalization with Benzimidazole Groups (NM_BENZ_/MSN_BENZ_)

The mesoporous face in MSNs and NMs were functionalized with benzimidazole groups to further react with β-cyclodextrins attached to ficin creating inclusion complexes that act as pH-triggered nanovalves. For that, 60 mg MSNs or NMs were suspended in 4 mL of anhydrous ACN and treated with 60 μL of (3-iodopropyl) trimethoxysilane for 5.5 h. Solids were isolated by centrifugation and washed with toluene. To prepare a saturated solution of benzimidazole, 0.25 g was mixed with 330 μL of TEA and 20 mL of toluene and heated at 80 °C until complete dissolution. Then, 10 mL of the benzimidazole solution was added over MSNs or NMs and the mixtures were magnetically stirred at 80 °C and refluxed for 3 days. Finally, the intermediate solids NM_BENZ_ and MSN_BENZ_ were centrifuged, washed with toluene, and dried at 37 °C.

#### Synthesis of β-Cyclodextrin-Modified Ficin (F-βCD)

25 mg of ficin was dissolved in 4 mL of 100 mM sodium phosphate buffer pH 6 at 4 °C and mixed with 21 mg of EDC and 21 mg of NHS for 30 min to activate the protein acid groups. Later, 13 mg of βCD-NH_2_ (synthesized as described previously)^55^ was added to the mixture, which was magnetically stirred for 12 h to generate amide bonds between the amino groups of the βCD-NH_2_ and the activated acid groups of the ficin protease. Finally, ficin conjugated with β-cyclodextrins (F-βCD) was dialyzed *vs* cold 100 mM sodium phosphate buffer (PBS), pH 7.5, using Amicon Ultra-05 centrifugal filter units (3 kDa).^56^ F-βCD was conserved at 4 °C until use.

#### Janus Pt–MSN Nanomotors Loaded with Vancomycin and Capped with Ficin (NM_VF_)

NMs nanopores were loaded with the antibiotic vancomycin. In a typical synthesis, 50 mg of NM_BENZ_ was mixed with 7.5 mg of vancomycin in 4 mL of 100 mM PBS pH 7.5 and stirred magnetically overnight. Then, the solid was isolated by centrifugation and resuspended again in PBS. To attach the molecular gate (F-βCD), vancomycin-loaded NM_BENZ_ was reacted with 12.5 mg of F-βCD at 4 °C overnight. Lastly, NM_VF_ was centrifuged and washed several times with 100 mM PBS pH 7.5.

#### Control Janus Pt–MSN Nanomotors Loaded with Vancomycin and Capped with Cyclodextrins (NM_V_)

To synthesize the control nanomotor without ficin, NM_V_, 15 mg of vancomycin-loaded NM_BENZ_ reacted overnight with 5 mg of β-CD in 3 mL of 100 mM PBS pH 7.5.

#### Control Janus Pt–MSN Nanomotors Capped with Ficin (NM_F_)

For the synthesis of the control nanomotor without vancomycin, NM_F_, 15 mg of NM_BENZ_ reacted with 3.75 mg of F-βCD in 3 mL of 100 mM PBS pH 7.5 overnight at 4 °C.

#### Control MSN Loaded with Vancomycin and Capped with Ficin (MSN_VF_)

The control nanodevice without PtNDs, MSN_VF_, was prepared following the same procedure described for NM_VF_, employing MSN_BENZ_ instead of NM_BENZ_.

#### Synthesis of Vancomycin–Rhodamine B Conjugate (V–Rh)

For UV–visible and CLM visualization purposes, vancomycin was modified with the dye rhodamine B (V–Rh).^57^ 5 mg of rhodamine B (10 μmol, 1.4 eq) was activated with an excess of EDC (15 mg, 115 μmol) and NHS (22 mg, 190 μmol) in 2 mL of 100 mM PBS pH 6 for 30 min. Then, the mixture was added to 10 mg of vancomycin (7 μmol, 1.0 equiv) and incubated at 4 °C overnight.

#### Janus Pt–MSN Nanomotors Loaded with Vancomycin–Rhodamine and Capped with Ficin (NM_V-RhF_)

Solid NM_V-RhF_ was prepared following the same steps employed for the synthesis of NM_VF_. However, their pores were loaded with V-Rh. All functionalized nanodevices were kept at 4 °C until use. Table S1 summarizes all of the nanodevices employed in the study.

#### NM_VF_ Peroxidase-like Activity Test

To evaluate the catalytic properties of NMVF, a peroxidase-like activity test was performed.^58^ The test is based on the platinum capability to oxidize ABTS to ABTS* in the presence of H_2_O_2_ (H_2_O_2_ + ABTS → ABTS* + H_2_O). ABTS* is a blue-colored product detectable by UV–visible spectrophotometry. Reactant concentrations were the following: 500 mL of ABTS (9 mM), 500 mL of H_2_O_2_ (from 0 to 30 mM), and 40 mL (1 mg mL^–1^) of NM_VF_. The variation of the absorbance (λ_abs_ = 405 nm) was kinematically recorded for 2 min at 25 °C. As can be observed in Figure S8, NM_VF_ owns an intrinsic peroxidase activity with a typical Michaelis–Menten behavior, where the kinetic parameters, Michaelis–Menten constant (*K*_M_), and the maximum velocity (*V*_max_) were estimated from the Lineweaver–Burk plot. Results were obtained by the application of eq 1.
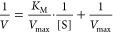
1where *V*_max_ is the maximum velocity, *K*_M_ is the Michaelis–Menten constant, and *S* is the concentration of the substrate.

#### Optical Video Acquisition and Motion Analysis by MSD Analysis

For the evaluation of the nanomotors’ motion capability, nanoparticle single tracking analysis (NTA) was applied. NM_VF_ were dispersed in 100 mM PBS pH 7.5 at a concentration of 0.002 mg mL^–1^, ultrasonicated, and introduced by a 1 mL syringe in the Nanosight NS300 device chamber (total volume 800 μL) at a temperature of 25 °C, in the presence of several concentrations of the fuel, H_2_O_2_ (0, 0.035, 0.105, and 0.2%, v/v). Fuel solutions were already present in the chamber when nanodevices were added, to avoid any drift interference. Next, 5 videos of 30 s at a speed of 30 frames s^–1^ were recorded by a sCMOS camera coupled to an optical microscope. The NTA 3.0 software was employed to analyze the x and y coordinates of 15 nanomotors (between 100 and 400 nm in size) over time (Δ*t* = 0.4 s), to calculate, by applying eq 1, the mean-square displacement (MSD) for each one of them. *D*_eff_ for nanomotors in the presence of H_2_O_2_ and *D*_0_ for nanomotors in the absence of H_2_O_2_ were obtained by plotting MSD *vs* Δ*t*, through the linear component of the Stokes–Einstein equation, eq 2, by an in-house developed R code.

In addition, NM_V-RhF_ movement in the presence of *S. aureus* cells was corroborated. 1 mg mL ^–1^ NM_V-RhF_ and 0.15% of H_2_O_2_ were incubated with a preformed 6 h old *S. aureus* biofilm in an IBIDI μ-Slide I Luer microfluidic device, and a 10 min time-lapse was recorded by CLSM (Video S1, green: rhodamine of NM_V-RhF_, red: matrix polysaccharide labeled with the Alexa Fluor 680-dextran conjugate).
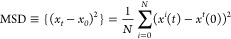
2where *N* is the number of averaged nanoparticles and *x*^*i*^ and *x*^*t*^ are the vector positions of particles at different times.
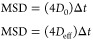
3where *D*_0_ is the diffusion coefficient of nanomotors without fuel and *D*_eff_ is the effective diffusion coefficient of nanomotors in the presence of fuel.
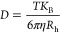
4where η is the viscosity, 10^–3^ Pa·s; *k*_B_ is the Boltzmann constant; *T* is the temperature, 25 °C; and *R*_h_ is the hydrodynamic radius, 191.35 nm.

#### Determination of β-CD Attached to Ficin

A typical phenol–sulfuric acid method for carbohydrate determination with some modifications was employed to calculate the quantity of β-CD attached to ficin.^59^ 100 mL of phenol (3% in DI water) was added to 100 mL of unmodified ficin or F-βCD (1 mg mL^–1^) and incubated for 10 min at 25 °C. Later, 500 mL of H_2_SO_4_ was added. Moreover, a β-CD calibration curve was prepared at concentrations of 350, 250, 150, 75, 30, and 20 μg mL^–1^, and the same procedure used for the modified enzyme was followed. The formation of a yellow-colored compound was determined by UV–visible spectrophotometry (λ_abs_ = 490 nm). β-CD bounded to ficin was calculated to be 4% by interpolation on the calibration curve.

#### Determination of the Quantity of Ficin on NM_VF_

For the determination of the NM_VF_ ficin enzyme content, the BCA method was applied following the procedure suggested by the provider with some adaptations.^60^ 200 mL of B/A solution (1:50) was mixed with 10 mL of NM_VF_ (1 mg mL^–1^) and incubated for 10 min at 60 °C. Then, samples were centrifuged at 4 °C, and the absorbance at 570 nm was measured. The calibration line was prepared with different concentrations of ficin (from 7.8 to 1000 mg mL^–1^) solved in 100 mM sodium phosphate buffer pH 7.5 following the same steps. Ficin attached to NM_VF_ was calculated to be 18.7 μg per mg of the nanomotor.

#### NM_VF_ Protease Activity Test

NM_**VF**_ protease activity was studied by a casein degradation assay.^36^ 1 g of casein was solved in 9.9 mL of 100 mM PBS pH 7.5 at 60 °C for 15 min to ensure complete dissolution. Then, 1 mL of 1% casein was mixed with 500 mL of NM_VF_ (1 mg mL^–1^) and incubated at 40 °C. 20 min later 3 mL of 5% trifluoroacetic acid was added to stop the reaction. Samples were centrifuged, followed by filtration (syringe nylon filter, 0.22 μm) of the supernatant. Finally, the protein concentration was measured by the BCA method.^60^ The same process was repeated for control samples and standards (casein, casein + free ficin, and ficin). The activity was calculated by applying the supplementary eq 5. NM_VF_ exhibited 1.02 × 10^–5^ U mg^–1^.
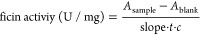
5where *A* is the absorbance (λ_abs_ = 570 nm), the slope is the slope of the calibration line, *t* is the incubation time with casein, and *c* is the concentration of the sample.

#### Controlled Cargo Release Experiments

To study the nanomotors’ capability to deliver vancomycin, 1 mg of NM_V-RhF_ was suspended in 1 mL of 100 mM PBS pH 7.5 (blank), and 1 mg of NM_V-RhF_ was suspended in 100 PBS pH 4.5. Mixtures were stirred in a shaker at 37 °C. At different times (0, 30, 60, 90, and 120 min), samples were centrifuged (5 min, 12.500 rpm, 4 °C) to remove NM_V-RhF_. Lastly, the fluorescence emission of the V–Rh released to the supernatant was measured (λ_exc_ = 546 nm, λ_em_ = 568 nm).

#### Bacterial Strains and Growth Conditions

Experiments were performed using *S. aureus* strain Sa240 (*S. aureusssp. aureus* Rosenbach 1884). The strain was plated on TSA and TSB and grown overnight at 37 °C with vigorous shaking at 120 rpm.

#### H_2_O_2_ Effect on Planktonic and Biofilm Growth

To assess the H_2_O_2_ effect on planktonic Sa240 growth, 100 μL (OD_600_ = 0.175) of the bacterial suspension was added into corresponding 96-well plates in triplicate for each condition. After that, 100 μL of H_2_O_2_ diluted in TSB supplemented with 0.25% of filter-sterilized d-glucose (TSB-glu) was added reaching final H_2_O_2_ concentrations of 0.35, 0.30, 0.25, 0.20, 0.15, and 0.1% (v/v). Continuous Sa240 growth was then monitored by means of absorbance at 610 nm for 24 h at 37 °C.

#### Real-Time Biofilm Eradication

To evaluate the effect of different nanoparticles on 6 h preformed *S. aureus* Sa240 biofilms, xCELLigence Real-Time Analyzer was used in accordance with the manufacturer’s instructions. The preformed biofilm eradication experiments were performed, as previously described,^61^ where biofilm growth was expressed as the cellular index (CI), which corresponds to a total biofilm mass. Briefly, *S. aureus* 240 was grown overnight in TSB media. One hundred microliters of TSB-glu were used for background measurements (in triplicate for each condition). After that, 75 μL of the Sa240 bacterial suspension diluted in TSB-glu to OD_600_ = 0.153 was added into the corresponding E-plate wells, reaching a final concentration of OD_600_ = 0.0875. This OD corresponds to approximately 1 × 10^7^ cells. Subsequently, E-plates were incubated in an xCELLigence system at 37 °C, and the biofilm growth was measured for 6 h with CI values registered every 10 min. After 6 h of biofilm growth, the experiment was stopped, and different treatments were added into the corresponding wells in E-plates. Following the treatments, biofilm growth was observed for an additional 24 h. Appropriate negative controls were included in each experiment in triplicate. CIs values (total biofilm mass) were obtained by subtracting their respective negative controls.

#### Viable Colony Count Assay

To assess the number of viable unattached planktonic bacteria after *S. aureus* Sa240 biofilm treatments, the supernatant was collected, and serial 10-fold dilutions were prepared. After that, 100 μL of each dilution was plated on TSA plates. Three biological replicates were plated for each condition. To assess the viable cell number in bacterial biofilms, the biofilms were rigorously rinsed using PBS pH 7.4 to eliminate nonadherent cells. After that, the biofilms were collected using 200 μL of PBS and sonicated for 5 min to disrupt the biofilm matrix and release biofilm-embedded *S. aureus* cells. Biofilms then were serial-diluted and plated in triplicate, as described above. TSA plates were incubated at 37 °C overnight. After that, viable colonies (CFUs) were counted, averaged, and expressed as log10.

#### Confocal Laser Scanning Microscopy

To show how nanoparticles interact with the biofilm EPS, the Alexa Fluor 488 fluorescent conjugate, which binds to *N*-acetyl neuramic acid and polysaccharide adhesin, involved in biofilm formation was employed. Briefly, *S. aureus* 240 biofilms were cultivated, by adding 175 μL of the bacterial suspension into the corresponding wells of IBIDI 80827 μ-Slide 8-well plates and biofilms, and biofilms were grown at 37 °C for 6 h as described above, in the presence of 50 μL of the Alexa Fluor 488 fluorescent conjugate (0.13 mg mL^–1^). Later, the supernatant was removed, and the biofilm was washed with TBS several times. After that 1 mg mL^–1^ NM_VF_, 0.15% H_2_O_2_ NM_VF_, and 0.15% H_2_O_2_ were added into the corresponding wells and Sa240 biofilms were grown for an additional 24 h.

To quantify live cells within bacterial biofilms after treatment with 1 mg mL^–1^ NM_VF_, 0.15% H_2_O_2_ NM_VF_, and 0.15% H_2_O_2_, the SYTO9 fluorescent dye that binds to microbial DNA was employed. Biofilms were grown, as described above. Thereafter, treatments were added into the corresponding wells, and Sa240 biofilms were grown for an additional 24 h. Subsequently, the supernatant was discarded, and the biofilms were carefully rinsed with PBS to eliminate unattached cells and stained with 200 μL of SYTO9 solution (working solution 3 μL of SYTO9 in 2 mL of sterile water) for 20 min in the dark. After biofilm staining, the excess dye was removed by gently washing using water. After that, CLSM image acquisition was carried out. To acquire the signals, a 679 nm excitation laser and 702 nm emission filters were used for the Alexa Fluor 680-dextran conjugate and a 488 nm laser and 505–550 emission filters were used for SYTO9.

#### Micromorphology of 24 h Biofilms after Treatment

To evaluate the biofilm spatial structure after the treatments, scanning electron microscopy (SEM) was performed. *S. aureus* 240 overnight culture was adjusted to OD_600_ = 0.0875, and the biofilms were grown on gold electrodes for 24 h. After that, biofilms were treated with NM_VF_ in the presence and absence of H_2_O_2_, VF, and V for 6 h. Then, supernatants were discarded, and biofilms were gently washed with PBS to eliminate nonadherent cells. Prior to observations, biofilm samples were fixed with Karnovsky’s fixative for 4 h, rinsed with PBS three times, and dehydrated using a gradual ethanol series (30–50–70%) twice. Then, the samples were dried using critical point drying with CO_2_. Subsequently, the samples were observed at SEM applying an accelerating voltage range of 0.5 kV and a magnification range of ×2.50 k.

#### Statistical Analysis

In order to study the differences between CIs, regression analysis was assessed by a linear model at 24 h of biofilm growth, using the lm library in the R Statistical Package version 1.0.7.1.^62^ Statistical differences in the viable cell number were assessed using Student’s *t*-test. The data presented as the mean ± SDs from triplicates of three independent experiments for each condition (*n* = 9). For comparisons between the means in CLSM analysis, ordinary one-way ANOVA and Dunnett’s multiple comparison tests were employed (*n* = 3). *p*-values < 0.05 were considered significant.

## Supporting Information Available

Description of the nanomaterials used in this study; PXRD patterns; N_2_ adsorption–desorption isotherms; TG analysis; hydrodynamic diameter; ζ potential; V–Rh fluorescence emission spectrum; peroxidase-like activity test; nanomotor trajectories; and control tests in bacterial culture (PDF)
Movement of NM_V-RhF_ (spherical shape) in the presence of planktonic *S. aureus* cells, *S. aureus* cells and NM_v-RhF_ were marked in green and EPS in red (Video S1) (AVI)

## Supplementary Material

cm3c00587_si_001.pdf

cm3c00587_si_002.avi
