# Supplementary material for: Ficin–Cyclodextrin-Based Docking Nanoarchitectonics of Self-Propelled Nanomotors for Bacterial Biofilm Eradication
Source: Chem Mater. 2023 May 9;35(11):4412–26. doi: 10.1021/acs.chemmater.3c00587 (PMC10269336; doi:10.1021/acs.chemmater.3c00587)
Supplement: Supplementary file 1 — cm3c00587_si_001.pdf [file cm3c00587_si_001.pdf]

Supporting Information

Ficin-Cyclodextrin-Based Docking Nanoarchitectonics of Self-Propelled Nanomotors for Bacterial Biofilm Eradication

Miglė Žiemytė, [a†] *Andrea Escudero,*[b,c,d†] *Paula Díez,*[b,c,e,f*] *María D. Ferrer,* [a,g] *Jose R. Murguía* [b,c,f]*, Vicente Martí-Centelles* [b,c]*, Alex Mira* [a,g*] *and Ramón Martínez-Máñez* [b,c,d,e,f*]

[a] Genomics & Health Department, FISABIO Foundation, 46020, València, Spain.

[b] Instituto Interuniversitario de Reconocimiento Molecular y Desarrollo Tecnológico (IDM), Universitat Politècnica de València, Universitat de València, Spain.

[c] Unidad Mixta UPV-CIPF de Investigación en Mecanismos de Enfermedades y Nanomedicina, València, Universitat Politècnica de València, Centro de Investigación Príncipe Felipe, 46012, València, Spain.

[d] Unidad Mixta de Investigación en Nanomedicina y Sensores. Universitat Politècnica de València, Instituto de Investigación Sanitaria La Fe, 46026, València, Spain.

[e] CIBER de Bioingeniería, Biomateriales y Nanomedicina (CIBER-BBN), Instituto Carlos III, 28029, Madrid, Spain.

[f] CIBER of Epidemiology and Public Health (CIBERESP), Instituto Carlos III, 28029, Madrid, Spain.

[g] Departamento de Química, Universitat Politècnica de València, Cami de Vera s/n, 46022, València, Spain.

*E-mail: paudiesa@upvnet.upv.es, mira_ale@gva.es, rmaez@qim.upv.es

Table S1. Description of nanomaterials used in this study.

Nanomaterial		Description
**NM_{VF}**	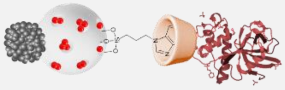	**FINAL DEVICE:** NMs loaded with vancomycin and capped with F-βCD
**NM_{V}**	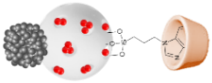	NMs loaded with vancomycin and capped with β-CD, without ficin
**NM_{F}**	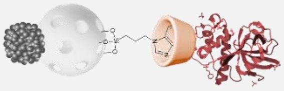	Empty NMs capped with F-βCD
**MSN_{VF}**	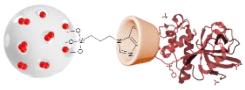	MSNs loaded with vancomycin and capped with F-βCD, without PtNDs
**$NM_{V\text{-}RhF}$**	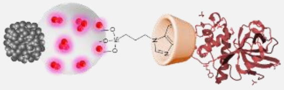	NMs loaded with V-Rh B and capped with F-βCD
**NA**	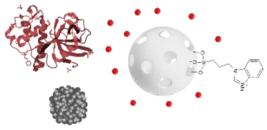	Not assembled nanomotor

- **Nanomaterials characterization**

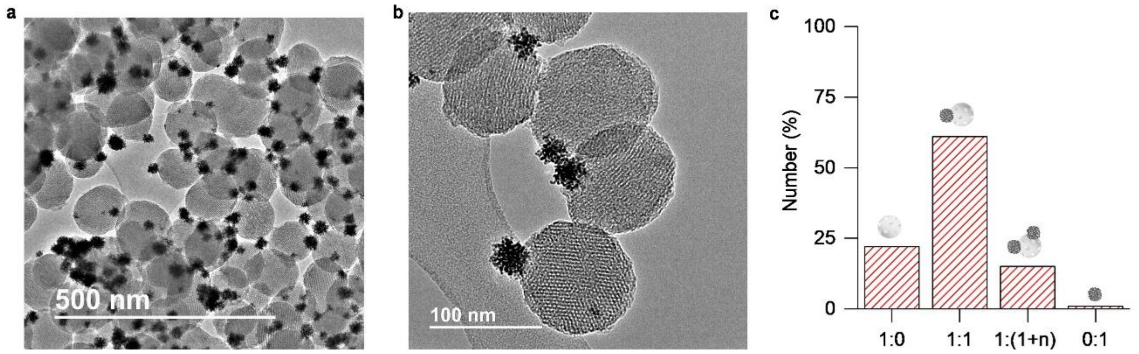

Figure S1. (a) HR-TEM image of Janus Pt-MSN nanoparticles (NMs). (b) Zoomed HR-TEM image of NMs, showing the typical porous hexagonal arrangement. (c) Efficiency

of the synthesis process, quantifying the number of nanoparticles obtained with different MSN:Pt ratio (1:0, 1:1, 1:(1+n), and 0:1), n = 160.

The synthesis efficiency of Janus Pt-MSN nanoparticles was evaluated by recording several HR-TEM images of the obtained sample. The result showed that the majority of nanoparticles (61%) was obtained in the preferred 1:1 ratio (MSN:Pt), while MSNs without Pt or those with several Pt nanoparticles represent 22% and 16%, respectively. A total of 160 nanoparticles were counted.

PXRD analysis

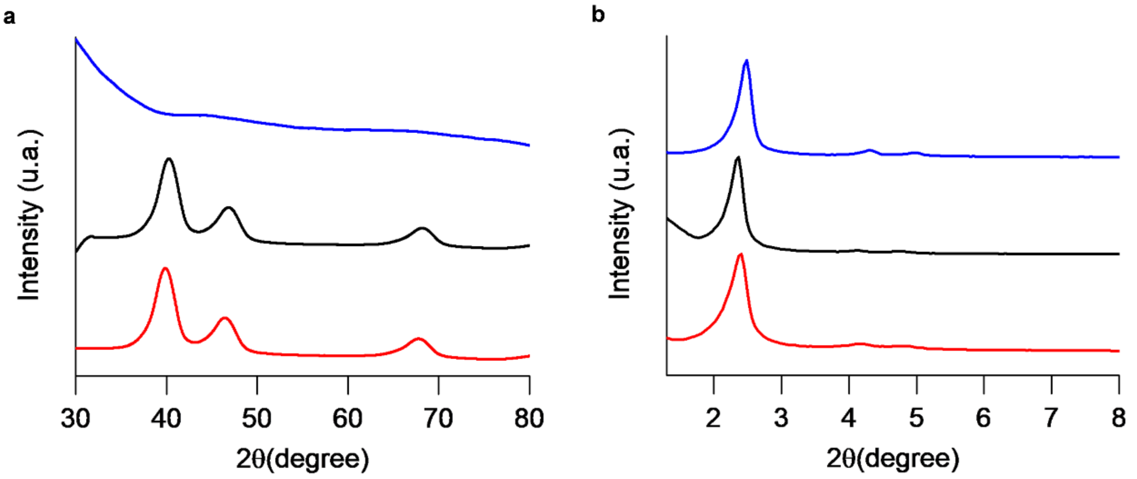

Figure S2. PXRD patterns at high (a) and low (b) angles of calcined MSNs (blue line), NMs (black line) and NM_{VF} (red line).

Data at low angles reflects the typical mesoporous structure in NMs through the main peak at 2.4°, which is also present in NM_{VF} showing that the surface functionalization and pores filling did not damage the mesoporous structure. Bragg peaks at high angles (111), (200) and (220) confirm the PtNDs cubic structure.

N_2 adsorption-desorption isotherms

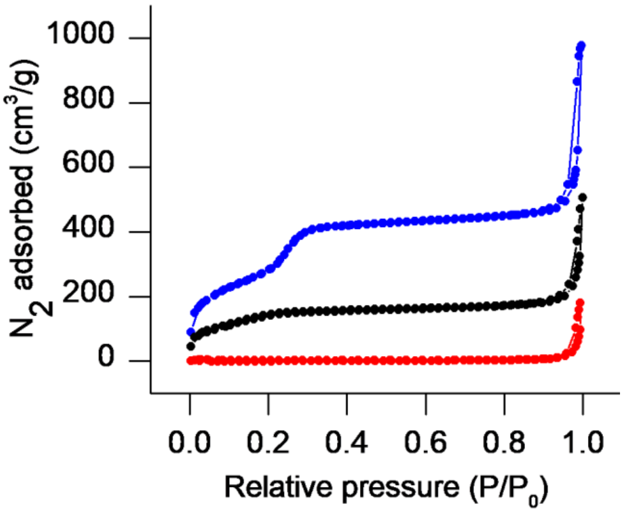

Figure S3. N_2 adsorption-desorption isotherms for MSNs (blue), NMs (black), and NM_{VF} (red).

The N_2 adsorption-desorption isotherms of the engineered MSNs and NMs show two main adsorption steps. The observed at an intermediate value, 0.3 P/P_0, is due to the N_2 condensation inside the mesopores by capillarity and reveals that the pores are empty. In addition, the absorption step at 0.9 P/P_0 corresponds to the interparticle porosity. N_2 adsorption-desorption isotherm for NM_{VF} reveals a great decrease the N_2 volume adsorbed, compared to those in MSNs and NMs, in agreement with pore loading with vancomycin and pore blocking with F-βCD. From this N_2 adsorption-desorption isotherms pore sizes, pore volumes and specific surface areas for MSNs, NMs, and NM_{VF} were calculated by applying BJH and BET models. Results are listed in **Table S2**:

Table S2. MSNs, NMs, and NM_{VF} pore diameters, pore volume (calculated by BJH model on the isotherm adsorption branch for $P/P_0 < 0.6$) and BET values.

	**BJH pore ($P/P_0 < 0.4$) (nm)**	**Total pore volume ($cm^3\ g^{-1}$)**	**BET ($m^2\ g^{-1}$)**
**MSNs**	2.2	1.0	1085
**NMs**	2.2	0.3	551
**NM_{VF}**	-	-	5.8

TGA

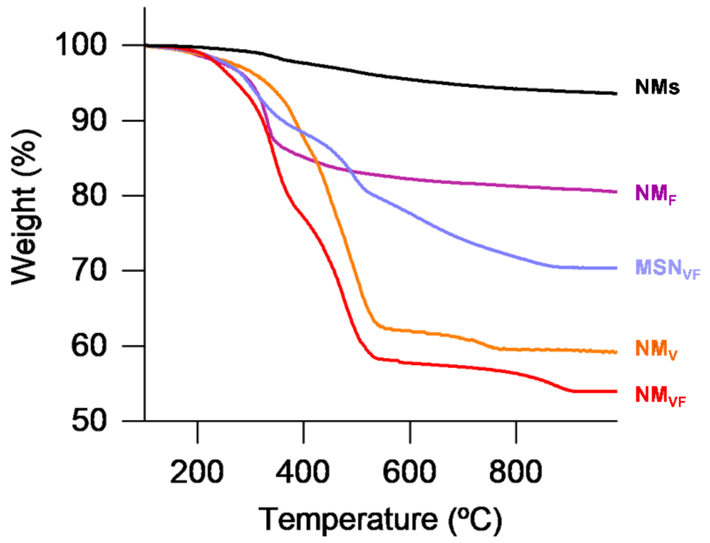

Figure S4. TGA of NMs (black line), NM_F (purple line), MSN_{VF} (blue line), NM_V (orange line), and NM_{VF} (red line).

From TGA analysis contents of the cargo[1] and the gatekeepers of the control nanomaterials synthetized were obtained. They are summarized in Table S3.

Table S3. Contents of gatekeeper and vancomycin *per* mg of solid for NM, NM_F, NM_V, MSN_{VF} and NM_{VF}.

**Nanomaterial**	**Gatekeeper ($\mu g\ mg^{-1}$)**	**Vancomycin ($\mu g\ mg^{-1}$)**
**NM**	-	-
**NM_F**	19.8	-
**NM_V**	16.6	22.3
**MSN_{VF}**	10.2	19.4
**NM_{VF}**	18.6	22.4

DLS and Zeta Potential analysis

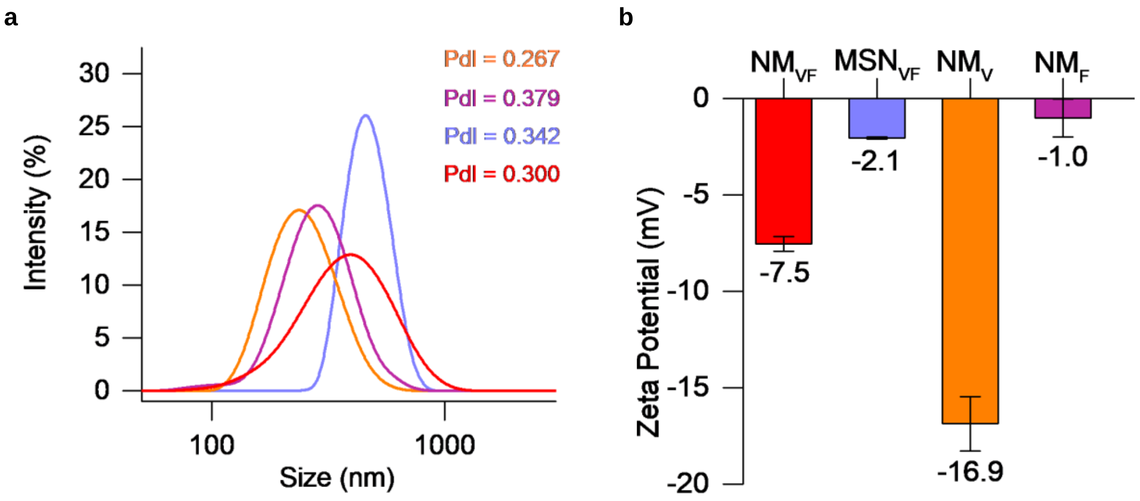

Figure S5. (a) DLS and (b) zeta potential analysis of control nanodevices, MSN$_{VF}$ (blue) NM$_V$ (orange) and NM$_F$ (purple), in comparison with NM$_{VF}$ (red).

The average hydrodynamic diameter of the control nanodevices was comparable to that observed for the final nanomotor NM$_{VF}$ (383 ± 7 nm). Regarding the superficial charge, all nanomaterials presented negative zeta potential values, especially NM$_V$ due to the absence of ficin.

Table S4. Summary of control nanomaterials size measured by DLS.

Nanomaterial	Size (nm)
**NM$_V$**	267 ± 23
**NM$_F$**	391 ± 15
**MSN$_{VF}$**	534 ± 8

- **On-command cargo-controlled release analysis**

V-Rh fluorescence emission spectrum

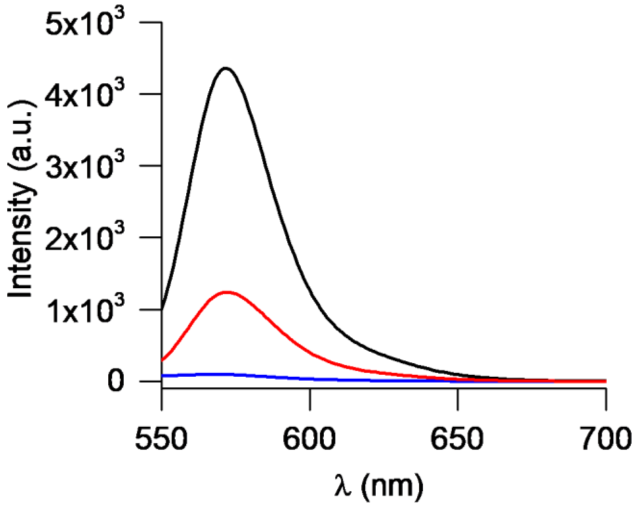

Figure S6. Fluorescence emission spectra of vancomycin (blue line), rhodamine B (black line) and vancomycin-rhodamine B conjugation (V-Rh) (red line) in 100 mM PBS (pH 7.5) (λ_{ex} = 556 nm). V-Rh showed the characteristic fluorescence of rhodamine B confirming the successful conjugation of rhodamine B to vancomycin.[2]

- **Motion study**

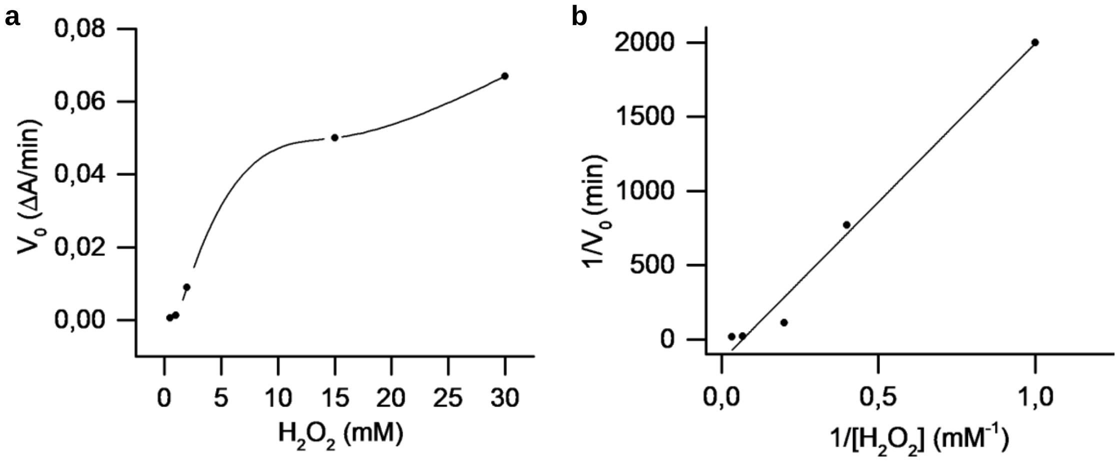

Figure S7. NM_{VF} peroxidase-like activity assay. (a) effect of H_2O_2 concentration on the nanomotor catalyzed reaction rate at the fixed ABTS dye concentration of 9 mM. (b) Lineweaver-Burk graph of H_2O_2-catalysed decomposition used to calculate K_M and V_{max} parameters applying the equation 1. Results were K_M: 14.95 mM and V_{max}: 6.99 µM min^{-1}.

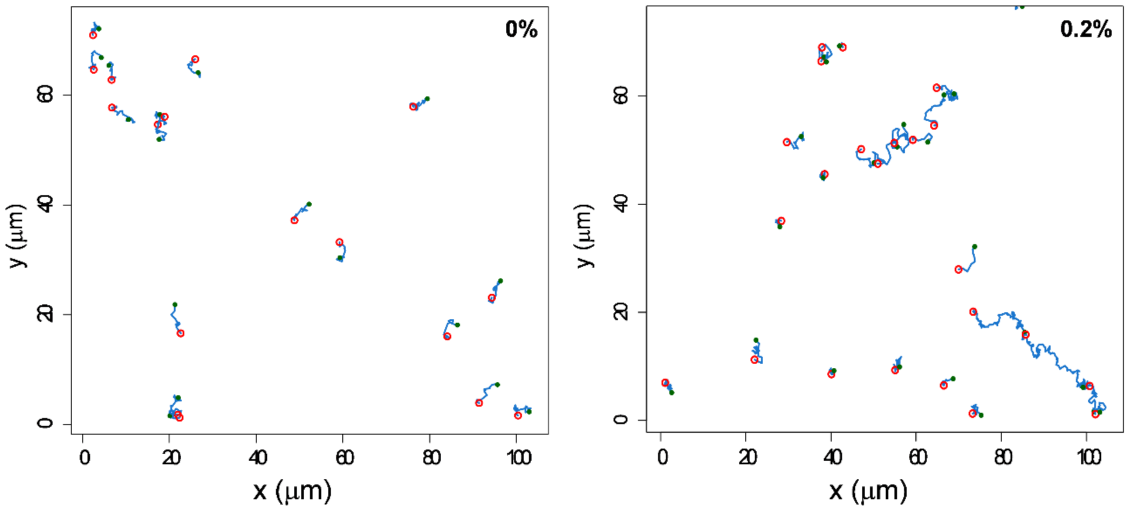

Figure S8. NM_{VF} trajectories recorded in the Nanosight NS300 sample chamber, extracted from one of 30 s videos at 0 and 0.2% of fuel. (Initial coordinates marked in red, final coordinates in green).

- **Eradication of pre-formed and mature *S. aureus* biofilms**

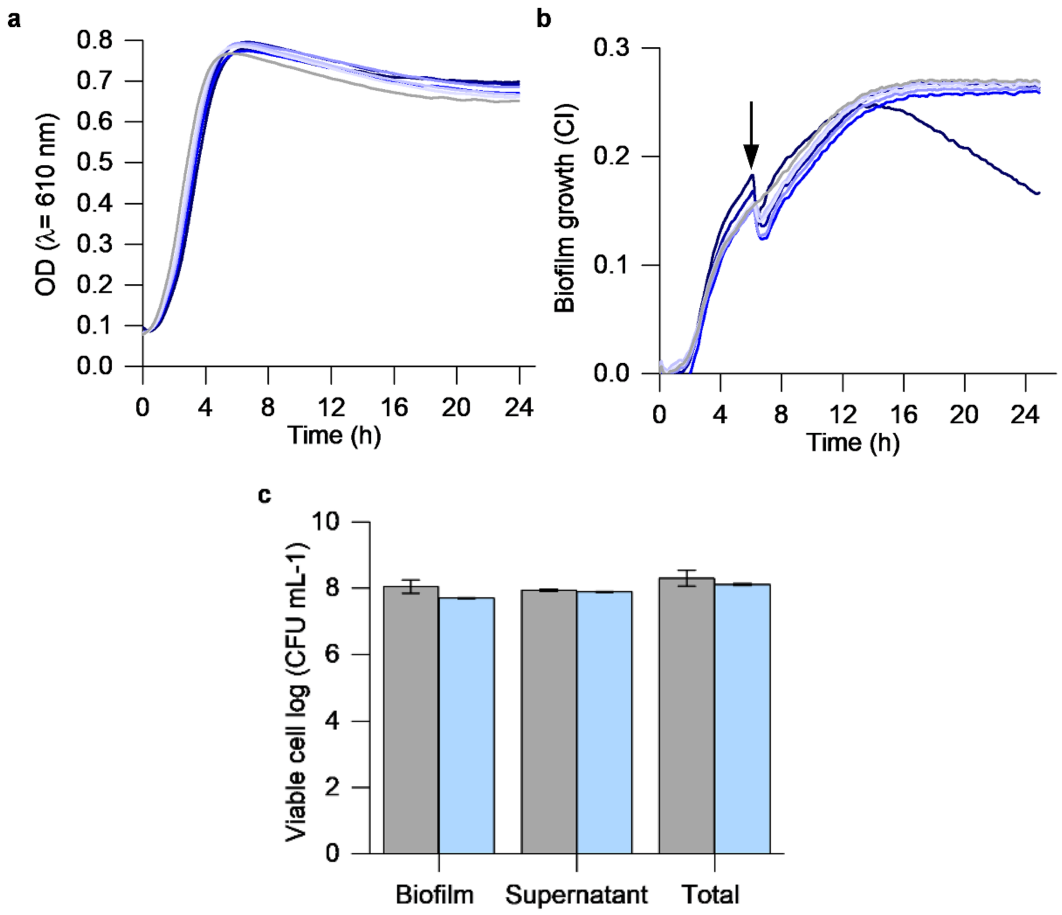

Figure S9. (a) Effect of different H_2O_2 concentrations: 0 (grey), 0.10, 0.15, 0.20, 0.25, 0.30 and 0.35% (from light blue to dark blue) on planktonic *S. aureus* growth, measured as optical density (OD) at 24h (H_2O_2 was added at initial time). (b) Effect of H_2O_2 on biofilm biomass of *S. aureus*, measured as CI impedance. Same concentrations of H_2O_2 were added at the exponential growth phase (marked by a black arrow). (c) Bacterial cell viability in biofilm and supernatant after addition of selected 0.15% of H_2O_2, expressed as log (CFU mL^{-1}) ± SE. (n=3, all total viable cell T-test values $p > 0.05$).

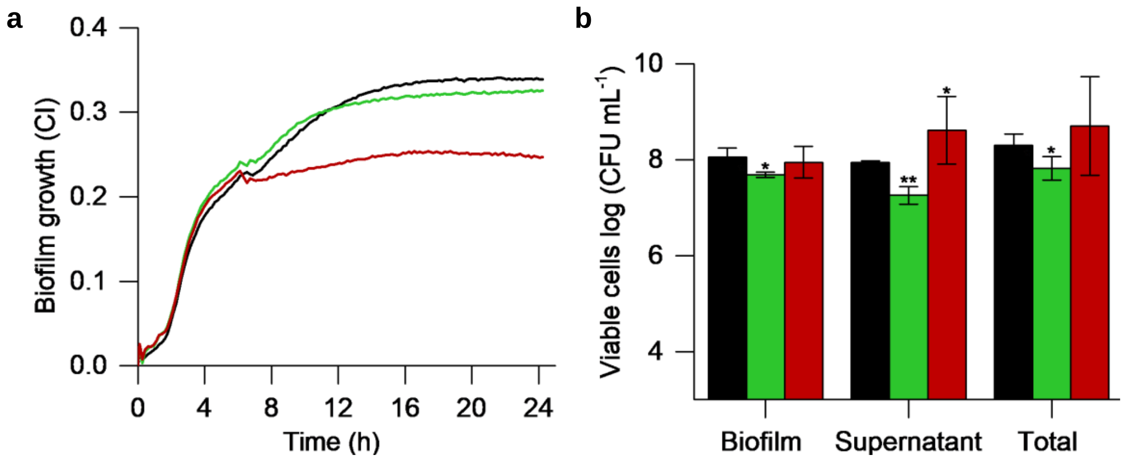

Figure S10. Comparative effect of F (red) and V (green) on pre-formed *S. aureus* biofilms at 24h. (a) Biofilm biomass (CI) (treatments were added at 6 h). (b) Bacterial cell viability in biofilms and supernatants after different treatments, expressed as log (CFU mL^{-1}). (n=3, T-test values $*p<0.05$, $**p<0.01$). Treatment concentrations: 23 μg mL^{-1} of V and 25 μg mL^{-1} of F. Free V and F only induced a reduction in the total biofilm mass of 4% and 27%, respectively, at 24 h.

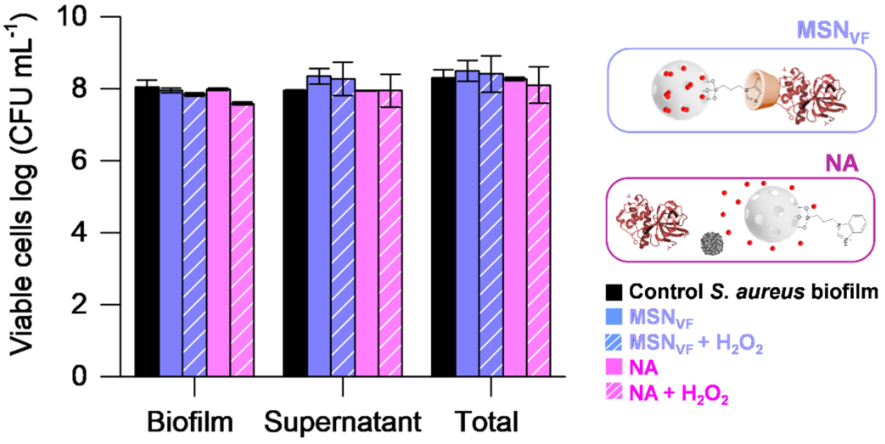

Figure S11. Bacterial cell viability in pre-formed *S. aureus* biofilms and supernatants after treatment with control MSN_{VF} (in the absence of H_2O_2, light blue/ in the presence of H_2O_2, stripped) and NA (in the absence of H_2O_2, pink/ in the presence of H_2O_2, stripped), expressed as log (CFU mL^{-1}). (n=3, all total viable cell T-test values $p > 0.05$). (Treatment concentrations: 1 mg mL^{-1} MSN_{VF}, 0.15% H_2O_2, and equivalent NA). The results showed that these controls did not induce bacterial cell death.

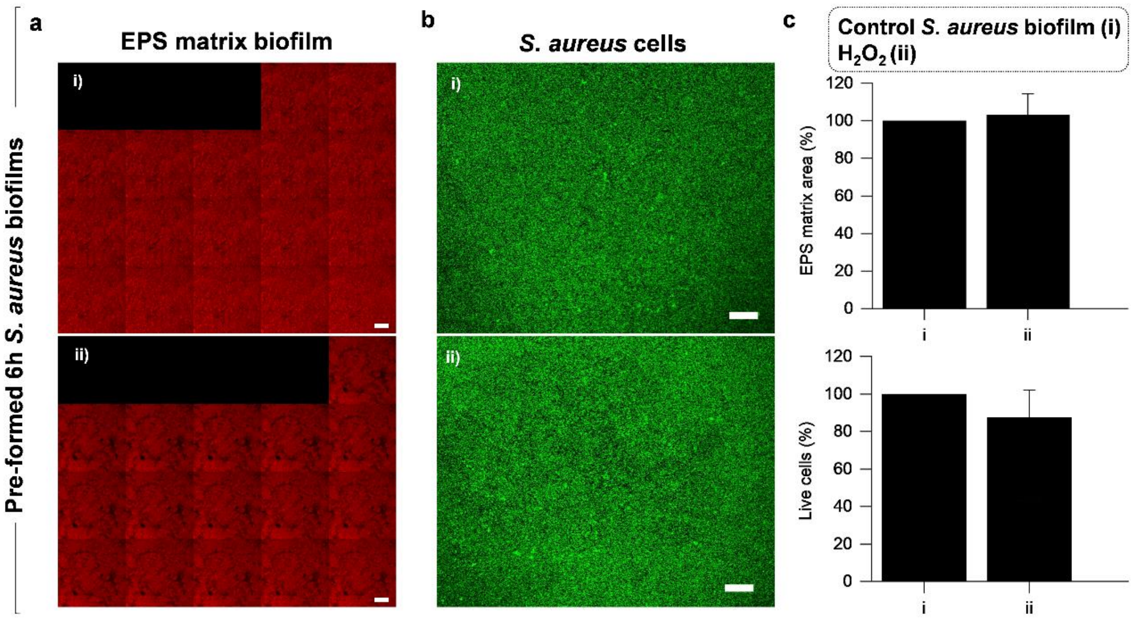

Figure S12. CLSM images showing the effect of 0.15% H_2O_2 (ii) on biofilm EPS disruption (a) and *S. aureus* cell viability (b). c Quantification of the area covered by EPS on the top layer of the biofilm and of the percentage of viable cells (data expressed as mean ± SE, n=3). Scale bar 200 μm.

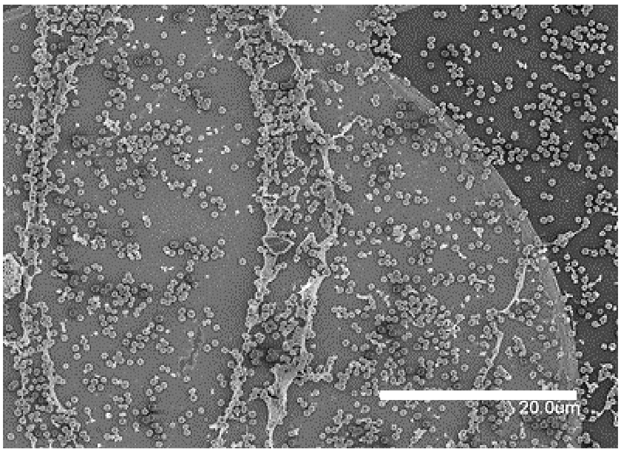

Figure S13. SEM micrograph showing the effect of the combination of V and F (23 and 25 μg mL^{-1}, respectively) on a 24 h mature *S. aureus* biofilm. Scale bar 20 μm.

References

(1) W. Zhang, R. Taheri-Ledari, Z. Hajizadeh, E. Zolfaghari, M. R. Ahghari, A. Maleki, M. R. Hamblin, Y. Tian, *Nanoscale* **2020**, 12, 3855-3870.

(2) C. C. Liu, L. S. Zhou, J. Y. Liu, J. M. Xiao, H. Z. Gao, K. W. Yang, *New J. Chem*. **2013**, 37, 575-580.
